# Functional traits, convergent evolution, and periodic tables of niches

**DOI:** 10.1111/ele.12462

**Published:** 2015-06-21

**Authors:** Kirk O. Winemiller, Daniel B. Fitzgerald, Luke M. Bower, Eric R. Pianka

**Affiliations:** ^1^ Program in Ecology and Evolutionary Biology and Department of Wildlife and Fisheries SciencesTexas A&M UniversityCollege StationTX77843‐2258USA; ^2^ Department of Integrative BiologyUniversity of TexasAustinTX8712‐0253USA

**Keywords:** Adaptive peak, bioassessment, ecological classification, ecological restoration, life history strategy, niche dimension, niche scheme, species ordination

## Abstract

Ecology is often said to lack general theories sufficiently predictive for applications. Here, we examine the concept of a periodic table of niches and feasibility of niche classification schemes from functional trait and performance data. Niche differences and their influence on ecological patterns and processes could be revealed effectively by first performing data reduction/ordination analyses separately on matrices of trait and performance data compiled according to logical associations with five basic niche ‘dimensions’, or aspects: habitat, life history, trophic, defence and metabolic. Resultant patterns then are integrated to produce interpretable niche gradients, ordinations and classifications. Degree of scheme periodicity would depend on degrees of niche conservatism and convergence causing species clustering across multiple niche dimensions. We analysed a sample data set containing trait and performance data to contrast two approaches for producing niche schemes: species ordination within niche gradient space, and niche categorisation according to trait‐value thresholds. Creation of niche schemes useful for advancing ecological knowledge and its applications will depend on research that produces functional trait and performance datasets directly related to niche dimensions along with criteria for data standardisation and quality. As larger databases are compiled, opportunities will emerge to explore new methods for data reduction, ordination and classification.

## Ecological Classification of Organisms: Towards a Periodic Table of Niches?

Nearing the end of his life, Robert MacArthur published a book chapter in which he made some predictions about the future of ecology (MacArthur 1972):

I predict there will be erected a two‐ or three‐way classification of organisms and their geometrical and temporal environments, this classification consuming most of the creative energy of ecologists. The future principles of the ecology of coexistence will then be of the form ‘for organisms of type A, in environments of structure B, such and such relations will hold.’ This is only a change in emphasis from present ecology. All successful theories, for instance in physics, have initial conditions; with different initial conditions, different things will happen. But I think initial conditions and their classification in ecology will prove to have vastly more effect on outcomes than they do in physics.

Building on MacArthur's proposal, Pianka (1974) appears to have been first to propose a *periodic table of niches*. He recognised that, given the multiple dimensions of the Hutchinsonian niche, creation of such a classification system would be difficult and necessarily would take a more complex, multidimensional form than chemistry's *periodic table of elements*. Here, we employ the term ‘dimension’ to mean a distinct aspect or facet of an entity or construct, as opposed to a physical or mathematical definition. Steffen (1996) suggested this analogy with chemistry is flawed because no set of functional characteristics could predict the ecological equivalent of chemical reactivity. Southwood (1977) and, more recently, Ferraro & Cole (2010) and Ferraro (2013) explored the related concept of *ecological periodic tables* based on the premise that habitat features provide the template for recurring properties of biotic communities. Despite considerable scepticism, the idea that such a classification system might be possible has remained in the literature. For example, McGhee's (2011) book on convergent evolution contains a catalogue of convergent phenotypes that spans diverse taxonomic groups. In the book's concluding chapter, McGhee briefly explores the idea of a *periodic table of life*:

Analogous to the ‘periodic table of niches’ … it is possible to create a ‘periodic table of life’ in a simple theoretical‐morphology thought experiment … We can use the chemical concepts of elemental complexity and evolutionary sequence in an analogous fashion by arranging the major groups of multicellular life in a similar series of rows of morphological complexity and biological evolutionary sequence … The columns of the periodic table can be considered to characterize the mobility of the elements in those rows …

At first glance, MacArthur's prediction seems not to have been realised; relatively few ecologists have pursued ecological classification systems of the sort he envisioned. A commonly held belief is that no general rules are possible in community ecology because of inherent complexity, prevalence of historical contingency, and large variation within study units ranging from populations to ecosystems. Given recognition that a strict analogy with chemistry's two‐dimensional periodic table of the elements is untenable, few ecologists have proposed methods for a niche classification scheme. We would argue that ecologists and natural resource managers, in fact, have already applied various niche classification schemes to natural resource management and environmental assessment. Ecologists frequently categorise species into functional groups based on certain aspects of the niche, while omitting, either advertently or inadvertently, other important niche dimensions that could enhance predictive power. For example, Azzurro *et al*. (2014) recently used external morphology to predict fish species potential for invasion success, but did not consider important niche dimensions, such as defence, physiology and life history.

Here, we revisit MacArthur's proposal for ecological classification, and propose that ecologists, often without realising it, have moved the needle well towards MacArthur's niche scheme, something akin to a periodic table of niches. Is this idea feasible, and could such a scheme prove useful for anything beyond vague heuristic purposes? Minimally, a standardised niche scheme, something analogous to a periodic table of niches, could provide a means to summarise and synthesise findings from disparate ecological classifications developed for diverse taxa, habitats and biomes. First, we contrast the idea of a periodic table of niches with chemistry's periodic table of elements, briefly reviewing efforts to arrange organisms from a functional traits perspective. We then explore the feasibility, limitations and a possible framework for development of such a scheme and identify some potential ecological applications. Our framework is based on five fundamental niche dimensions. Analysis of functional trait and ecological performance data sets associated with separate niche dimensions can produce either a continuous niche ordination scheme or a discrete niche classification scheme. To illustrate the potential of these approaches, we analysed a data set compiled for a tropical freshwater fish community. Our goal is to identify fundamental issues that merit further study to make niche schemes more operational, objective and broadly applicable.

## Periodic Tables of Elements and Niches

Dmitri Mendeleev (1869) created the periodic table of elements by organising known elements into rows and columns according to atomic weight and chemical reactivity. This organisation allowed him to realise that his periodic table was incomplete, and enabled him to make clear predictions about elements yet to be discovered. An analogous periodic table of niches would depend on the existence of *periodicity*, which would be a function of the degree to which species cluster around adaptive peaks defined by sets of trait combinations associated with certain environmental conditions. Ecology recently has seen a reawakening of interest in studying species assemblages from a functional traits perspective as opposed to a strictly taxonomic approach (Mouillot *et al*. 2013; Verberk *et al*. 2013). Functional traits have been used to predict spatial patterns of insect and fish species diversity, effects of disturbance on plant and fish communities and the influence of herbivores and seed predators on plant fitness among other things (Appendix S1). Public databases of species functional traits have been developed recently to support both basic and applied ecological research (e.g. Frimpong & Angermeier 2009; Kattge *et al*. 2011), making data required for construction of niche classification schemes more readily available. Westoby *et al*. (2002) reviewed efforts of plant ecologists to develop plant strategy schemes based on functional traits and ecological performance. They proposed a scheme that could assimilate information from worldwide research on plant ecology using a handful of traits and performance measures associated with four dimensions of ecological variation.

### Arguments against a periodic table of niches

Ecology faces challenges not shared by chemistry that complicate attempts to create similar classifications. Organisms and their habitats reveal variation across multiple dimensions and scales, are subject to stochastic influences, and contemporary observations are, to varying degrees, influenced by historical and geographical contingencies. This variation is perhaps the principal argument against a periodic table of niches, and also has contributed to a pessimistic view of general theories in ecology. Defining objective boundaries for ecological units of study is a universal challenge. For example, boundaries between different ecosystems, food web modules, populations and even species are often blurry or subjectively drawn. This means that two separate researchers attempting to build a periodic table of niches would achieve different results depending on how they chose to classify and organise this complexity. Another major challenge is niche multidimensionality and the need to develop methods to identify key niche dimensions and associated functional traits that allow for successful ecological predictions (Westoby *et al*. 2002; Laughlin 2014a).

Another argument against a periodic table of niches is the idea that species can evolve rapidly (Holt 2009), making entries into any classification scheme potential moving targets. Yet, a scheme that interprets adaptive peaks in terms of functional traits could benefit research on niche evolution by providing testable hypotheses of how species respond to changing abiotic and biotic environments. Such niche schemes also could facilitate investigations of adaptive radiation, niche conservatism, niche shifts during ontogeny and differential niche expression in relation to habitat, geography and community assembly (Colwell & Rangel 2009).

How then does one identify key attributes of niches? Proton number is constant among isotopes of a given element. Trait combinations, however, may not be constant for a given niche category, and most traits vary continuously rather than in a discrete manner. Statistical ordination methods have been used extensively to arrange organisms along environmental gradients, and sometimes have been used to infer adaptive strategies defined by trait combinations. Analysis of traits (variables) of organisms (observations) among various species assemblages (populations of observations) identifies gradients in trait space and allows ordination of organisms within that space (Lavorel *et al*. 2007; Mouillot *et al*. 2013). A variety of multivariate statistical methods have been developed to derive correlations between organisms and gradients of species assemblages with gradients of trait combinations and gradients of associated sets of variables describing habitats, regions or phylogenetic relationships (Dray & Legendre 2008; Kleyer *et al*. 2012; Laughlin & Laughlin 2013). Such methods identify constraints among all possible trait combinations and reveal greatly reduced numbers of what have been termed ‘functional trait niches’ (Poff *et al*. 2010).

### Arguments for a periodic table of niches

**Figure 1:**
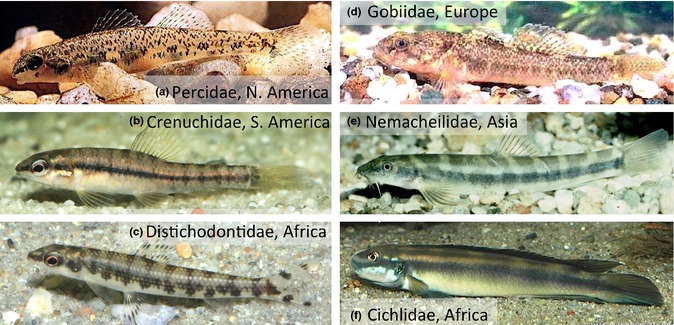
An example of globally distributed, strong evolutionary convergence – small fishes with cylindrical bodies and reduced swim bladders that rest upon sand or gravel in streams where they feed on benthic invertebrates, (a) *Etheostoma nigrum* Rafinesque (Percidae, North America), (b) *Characidium fasciatum* Reinhardt (Crenuchidae, South America), (c) *Nannocharax fasciatus* Günther (Distichodontidae, Africa), (d) *Padogobius nigricans* (Canestrini) (Gobiidae, Europe), (e) *Nemacheila notostigma* (Bleeker) (Nemacheilidae, Asia) and (f) *Gobiocichla wonderi* Kanazawa (Cichlidae, Africa). Photos courtesy of David McShaffrey (a), Massimo Lorenzoni (d) and Anton Lamboj (b, c, e and f).

A strong argument supporting the concept of a periodic table of niches is convergent evolution. Repeated patterns among form‐function relationships across divergent lineages (Fig. 1) represent clustering around adaptive peaks within the selection landscape, and periodicity in niche space. Despite unquestionable variation in ecological systems at all levels of organisation, some undeniable patterns among traits and trait combinations influence how organisms cope with their environments as well as how environmental features influence community assembly. Clearly, convergence is not all or none, present or absent, but exists along a gradient influenced by the degree of (1) functional trait similarity, (2) resolution used to measure traits, (3) lineage divergence among organisms being compared and (4) trait divergence that occurred prior to evolution towards similar functional traits. Yet remarkably similar designs appear throughout the animal kingdom (Conway Morris 2003; McGhee 2011). For example, Emlen (2008) reviewed structures used as weaponry in animal groups ranging from arthropods to dinosaurs and mammals, and Zakon (2002) examined convergent evolution at the molecular level, including opsins, gap junction proteins, neurotransmitter receptors and ion channels.

A single trait might show convergence merely due to random effects, however convergent evolution among suites of traits strongly implies determinism. Many traits have well‐known functions and vary predictably in relation to environmental features (Segar *et al*. 2013). Recent research has demonstrated functional trait convergence at the community level for diverse groups, including vascular plants (Sage 2004), beetles (Inward *et al*. 2011), fish (Ibañez *et al*. 2009) and lizards (Harmon *et al*. 2005; Mahler *et al*. 2013). The ubiquity of evolutionary convergence suggests that a general niche scheme is feasible, however, the scale and resolution for functional traits, ecological performance and phylogenetic relationships would determine its structure. Without convergence, no periodicity exists, and each species, or perhaps even each organism, is viewed as occupying its own unique niche. Of course, many potential trait combinations defining convergent niches will be missing within some taxonomic groups and species assemblages due to historical contingencies. Through evolution, dispersal and extinction, certain functional groups are gained and lost within lineages over time and space. For example, the fossil record reveals that dinosaurs and other archosaurs underwent adaptive radiations (Brusatte *et al*. 2008), and some modern day representatives (birds) occupy new regions of morphological space, whereas others (crocodiles) occupy only a small subset of their former niche space. In general, more diverse niches and fewer gaps within the total realm of niche space would be expected in diverse tropical communities than in those from similar habitats at higher latitudes containing fewer species.

Even if convergence fosters confidence that a periodic niche scheme is possible, we are still confronted with the problem of ecological complexity. One way to deal with ecological complexity is to adopt an approach similar to the one employed in chemistry. In the same way, elements can have different isotopes (varying atomic masses, but with essentially the same chemical properties), a niche category could have phenotypic variants but still have ecological properties or functions that are essentially the same. The periodic table of elements is a concise summarisation that ignores subatomic features with little relevance for basic chemical reactivity. Ecologists deal with substantially greater variation than chemists, but could aspects be defined in a manner similar to Mendeleev's scheme? The goal of such a scheme would not be the full description of biological diversity from genes to ecosystems, but rather the organisation of species in a way that can predict ecological responses, such as invasion success or population persistence within various environmental settings, or ecological effects, such as bioturbation or nutrient cycling.

**Figure 2:**
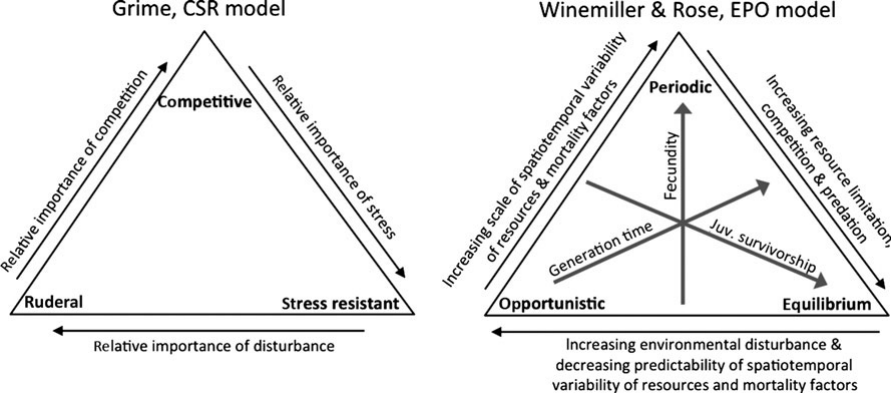
Comparison of the *C‐S‐R* and *E‐P‐O* life history models. Life history strategies comprise a fundamental niche dimension that can be defined by patterns of covariance, including those determined by constraints among functional traits associated with reproduction, growth and relative allocations of energy, biomass and time that evolve in response to selection.

Another argument in support of periodic tables of niches is the fact that ecologists have already identified patterns of trait covariation that are strongly and consistently associated with environmental gradients (Westoby *et al*. 2002; Poff *et al*. 2010; Smith *et al*. 2013). To illustrate this, we focus for a moment on life history strategies. Life history strategies identify suites of intercorrelated functional traits and their associations with patterns of environmental variation involving aspects such as physiological stress, temporal variation in environmental harshness, resource availability and quality, population density, risk of predation or parasitism and challenges for dispersal (Box 1). Organisms and species can be arranged within a life history surface or space defined by correlations among key traits that define allocation strategies (Fig. 2). Constraints among reproductive and demographic variables produce consistent syndromes, or strategies, and a strong basis for testing life history theories about selection. Life history is a strong candidate to be one of the fundamental niche dimensions for constructing a periodic table. What other dimensions should be included?

Box 1 **Life history strategies: functional traits ordination within a fundamental niche dimension**

Two life history schemes that predict adaptive evolution of suites of functional traits in response to selection imposed by abiotic and biotic environmental factors are the *Competitors‐Stress tolerants‐Ruderals* model originally based on insects and plants (Grime 1977, 1979; Southwood 1977), and *Equilibrium‐Periodic‐Opportunistic* model originally based on fishes (Winemiller 1992; Winemiller & Rose 1992). Both have endpoint strategies associated with colonising vs. competitive ability (*r* vs. *K* strategies respectively), but they differ in terms of environmental gradients selecting for endpoint strategies and the suite of attributes associated with the third strategy. The *C‐S‐R* model distinguishes a stress‐tolerant strategy in response to stressful environmental conditions (e.g. deficit of water or nutrients) that select for a suite of functional traits described as ‘beyond *K*’. In contrast, the *E‐P‐O* model identifies a periodic endpoint strategy characterised by long lifespan, high fecundity, periodic reproduction and low investment in individual propagules favoured in habitats with large‐scale environmental variation that influences early life stage survival (sometimes called ‘bet hedging’). Many trees, invertebrates and fishes would be classified as periodic strategists that reveal large interannual and spatial variation in recruitment.

The *C‐S‐R* model has proven useful for interpreting local species assemblage patterns in plants and other groups, including soil invertebrates and corals (Darling *et al*. 2012). At the same time, tests of the model have been inconclusive (Wilson & Lee 2000). Westoby (1998) pointed out that strategies within the *C‐S‐R* model are conceptual, and consequently plant species are not easily ordinated within the triangle. Westoby created the *Leaf‐Height‐Seed* model to permit species to be positioned within the scheme based on just three variables: specific leaf area, height of the plant's canopy at maturation and seed mass. The *L‐H‐S* scheme partially explained responses of vegetation communities to grazing in experiments (Moog *et al*. 2005; Golodets *et al*. 2009). The *E‐P‐O* model has been applied mostly to fishes, a group that reveals extreme variation in life history attributes relative to other animal groups (Winemiller 1992). The model has predicted significant variation in fish community structure in relation to patterns of streamflow (Mims & Olden 2012; Keck *et al*. 2014), landscape connectivity (Miyazono *et al*. 2010), harvest (Rose *et al*. 2001) and exotic species invasion (Olden *et al*. 2006). Flowering plants and arthropods are other groups that span large areas within the *E‐P‐O* continuum, but other groups, such as birds and mammals, occupy small zones (Winemiller 1992). Life history strategies reveal extensive convergence (from microbes to plants, invertebrates, fungi and vertebrates), and trait combinations that define how organisms allocate time, energy and biomass to reproduction to maximise fitness under different environmental conditions represent a basic niche dimension.

## How many Niche Dimensions are Sufficient?

The periodic table of elements has only two dimensions, and yet this scheme has tremendous utility in chemistry. Can a limited number of relevant niche dimensions be identified that will allow ecologists to make similarly useful predictions? To examine this question, Laughlin (2014a) created two data sets for five hypothetical species, a data set for correlated traits and another for uncorrelated traits, and estimated probability density functions for species using discriminant analysis based on Gaussian finite mixture modelling. Correlated traits reflected a single dimension and revealed limited interspecific niche differences; ordination based on uncorrelated traits reflecting more niche dimensions revealed greater ecological differentiation. When intrinsic trait dimensionality is higher, models more effectively reveal species differences within trait space and allow predictions of species distribution and abundance. Laughlin proposed that different sets of environmental filters select for independent trait dimensions. His analysis of three large empirical data sets of plant traits showed that ability to predict local species assemblage composition increases rapidly with number of traits, but reaches an asymptote with 4–8 traits.

In fact ecologists, either by intuition or logical deduction, have tended to focus research on a limited number of basic niche dimensions. Each of these dimensions is associated with ecological strategies defined by trait/performance combinations, and dimensional strategies often have associated sub‐strategies, most of which are fairly apparent (Table 1, see also Pianka 1993). Does a natural hierarchy of organisation exist among sets of constrained functional traits that have been moulded by natural selection? Let us consider the basic challenges confronted by all living organisms.

**Table 1:** Five niche dimensions with primary and secondary strategies and examples of ordination schemes or theories (Full references appear in Appendix S25)

Niche dimensions	Strategies	Examples	References
Habitat	Primary		
	Response to abiotic gradients	Species distribution and climate envelope models involving moisture, temperature, salinity, pH, etc.	Ferraro (2013), Negret *et al*. (2013), Pyke *et al*. (2013), Buckley *et al*. (2014)
	Secondary		
	Spatial	Migration, territoriality, sedentary/mobile, depth	Pianka (1966), Roff & Fairbairn (2007), Bentlage *et al*. (2013)
	Temporal	Diapause, hibernation, diel and seasonal activity	Danks (1987), Villegas‐Amtmann *et al*. (2013)
	Structural	Adaptation to substrates, structural complexity, substrate roughness	MacArthur & MacArthur (1966), Kolde *et al*. (2012)
Life History	Primary		
	Life history strategies	C‐S‐R, E‐P‐O and L‐H‐S models	Grime (1977), Winemiller & Rose (1992), Westoby (1998)
	Secondary		
	Temporal	Semelparity/iteroparity	Orzack & Tuljapurkar (1989)
	Physiological	Reproductive modes/guilds	Balon (1975), Chao *et al*. (2013)
Trophic	Primary		
	Feeding guilds	Animal trophic/feeding guilds, microbe/plant stoichiometry	Elser *et al*. (2000), Albouy *et al*. (2011), Rosas‐Guerrero *et al*. (2014)
	Secondary		
	Physiological	Nutrition/energy storage	Shertzer & Ellner (2002)
	Behavioural	Ambush vs. active search, spatial/temporal segregation, symbiosis	Pianka (1966), Villegas‐Amtmann *et al*. (2013), Chao *et al*. (2013)
Defence	Primary		
	Avoidance/resistance strategies	Fight or flight	Vanak *et al*. (2013)
	Secondary		
	Quantitative/qualitative	Theory of plant apparency	Feeny (1967), Massad *et al*. (2011)
	Mechanical/allelochemical	Weapons, chemicals, armour	Emlen (2008), Moles *et al*. (2013)
Metabolic	Primary		
	Metabolic rate strategies	Slow vs. fast metabolism	Brown *et al*. (2004), Humphries & McCann (2014)
	Secondary		
	Energy allocation	Acquisition vs. conservation, leaf economics	Shertzer & Ellner (2002), Wright *et al*. (2004), Buckley *et al*. (2014)

For any organism to survive, it must occupy a suitable abiotic environment with conditions within its tolerance limits. More often than not, the organism inhabits an environment that maximises fitness or surplus energy relative to metabolic demands (Buckley *et al*. 2014). Adaptation to habitat as influenced primarily by abiotic environmental characteristics, including structural complexity provided by vegetation, is the basis for the Grinnellian niche concept, the foundation for climate envelope models, or niche models, used to predict species geographic distributions within variable climatic and landscape scenarios (Elith & Leathwick 2009; Soberón & Nakamura 2009). This Grinnellian aspect of the niche could be called the *habitat dimension*, and provides a logical starting point for building a niche scheme based on functional traits and performance measures to predict how species respond to environmental gradients. Functional traits and performance measures involved in the habitat dimension would include performance/tolerance with respect to abiotic factors, such as temperature, moisture, pH, dissolved oxygen, salinity, toxic substances, etc. as well as the means by which organisms respond to gradients of structural complexity. Aquatic organisms physiologically adapted for salinities of marine vs. freshwater ecosystems provide a simple example of adaptation to abiotic factors, and numerous others could be cited (e.g. plant adaptation to moisture or soil gradients of moisture, pH, nutrients, etc.). Simple examples for adaptation to habitat structure are animals that require certain kinds of substrates for probing and extracting invertebrate prey: fishes (fishes from diverse taxa that have tube snouts and others that scoop and sift substrate within the orobranchial chamber), birds (woodpeckers, shorebirds) and mammals (armadillos, aardvarks, pigs). For most kinds of organisms, we already have identified numerous functional traits and performance measures that directly influence fitness along habitat gradients defined according to abiotic and structural environmental features.

As previously noted, life history strategies influence demographic responses to environmental variation, and therefore constitute a fundamental niche dimension. Successful reproduction is key to Darwinian fitness, and the extensive theoretical literature on life history strategies assumes limited solutions to environmental challenges (i.e. adaptive peaks). Functional constraints define these solutions, and extensive convergent evolution is therefore anticipated, and indeed is observed. Full development of a *life history dimension* might produce a hierarchical scheme involving demography (i.e. primary strategies, Box 1), energy/biomass allocation (e.g. reproductive effort, investment in individual offspring), reproductive timing (including diapause strategies), migration, etc. (Table 1).

A nutritional or *trophic dimension* also would be fundamental, because all organisms must acquire and assimilate resources for maintenance, growth and reproduction. Trophic guilds are one of the most intuitive concepts for grouping animals according to functional similarity. Trade‐offs involved in feeding mechanics (e.g. suction vs. raptorial feeding by fishes) and foraging strategies (e.g. sit‐and‐wait ambushers vs. active wide‐ranging searchers) has produced extensive convergent evolution among trophic niches in most major animal groups, and stoichiometric gradients have been used to arrange plants, microbes and herbivorous insects within nutritional niche space (Wakefield *et al*. 2005; Behmer 2009).

A survival or *defense dimension* would identify strategies for reducing mortality or damage caused by microbes, parasites and predators. The theory of plant apparency is one such scheme that identifies trade‐offs among qualitative and quantitative defences against herbivores (Feeny 1976), and basic structures used as weapons are shared by diverse animal taxa (Emlen 2008). The defence dimension also could contain multiple components, such as spatial (migration) vs. temporal (diel activity patterns, diapause) strategies to escape enemies.

A physiological or *metabolic dimension* would arrange organisms according to allocation strategies, such as energy conservation with low energy demand vs. high performance with high demand. Metabolic strategies are defined by fundamental bioenergetic constraints (Humphries & McCann 2014). Physiological trade‐offs involving respiration and assimilation of water and nutrients are a major focus in vegetation ecology (Reich *et al*. 2006), and physiological data associated with these and other performance trade‐offs are available for animals (thermoregulation, salinity tolerance, hypoxia tolerance, water conservation mechanisms, endurance, etc.) and microbes (physical and chemical tolerances/optima, nutrient assimilation, etc.).

Additional niche dimensions could be proposed, but we suggest these five are fundamental for any attempt to construct a periodic table of niches, and that addition of too many dimensions would limit ability to discern general patterns. No single dimension could capture the entire suite of traits and associated trade‐offs required to make accurate predictions about species response to environmental change and community assembly or stability (Adler *et al*. 2010). For example two species could have virtually identical trophic niches, body size and means of locomotion, and yet have different tolerances to abiotic conditions based on traits associated with the metabolic dimension. A niche classification scheme organised according to five dimensions could improve predictions over approaches focused on a single dimension (e.g. predictions derived from species distribution models, life history strategies or trophic guilds.) To differentiate niches of widely divergent life forms (e.g. microbes vs. vascular plants vs. metazoans), different kinds of traits and performance measures associated with certain niche dimensions would need to be emphasised. For example, the metabolic dimension has been the focus of ecological differences among bacteria and plants, and biochemical traits strongly influence habitat, trophic and defence dimensions for these groups. Animal ecology has tended to emphasise habitat and trophic niches, but integration of additional niche dimensions could improve predictive capabilities. Some herbivorous insects are trophic generalists in terms of habitat and diet, but the trophic niche dimension is partitioned on the basis of nutritional features of plants and plant parts, an interaction between the metabolic and trophic dimensions (Behmer 2009). For animal species coexisting within the same habitat, niche differences might involve some combination of strategies for habitat use, feeding, time and energy allocation and defence.

Given that the organism ultimately defines its niche, we recognise that our five basic niche dimensions will have some constituent traits in common. For example, body size is integral to multiple dimensions, and diel activity can be an essential component of strategies for habitat use, feeding, defence and metabolism. Behavioural traits could pose special challenges for any niche scheme, because behaviour is difficult to quantify, and can influence the adaptive value of morphological and physiological traits (e.g. thermoconformers vs. thermoregulators). Morphology sometimes fails to match predictions about performance due to behavioural plasticity or many‐to‐one mapping whereby different morphologies are functionally similar. In addition, behavioural and biochemical traits influencing and responding to indirect species interactions, such as facilitation, could be difficult to incorporate into a multidimensional niche scheme.

## What would the Ecological Analogue of a Periodic Table Look Like?

**Figure 3:**
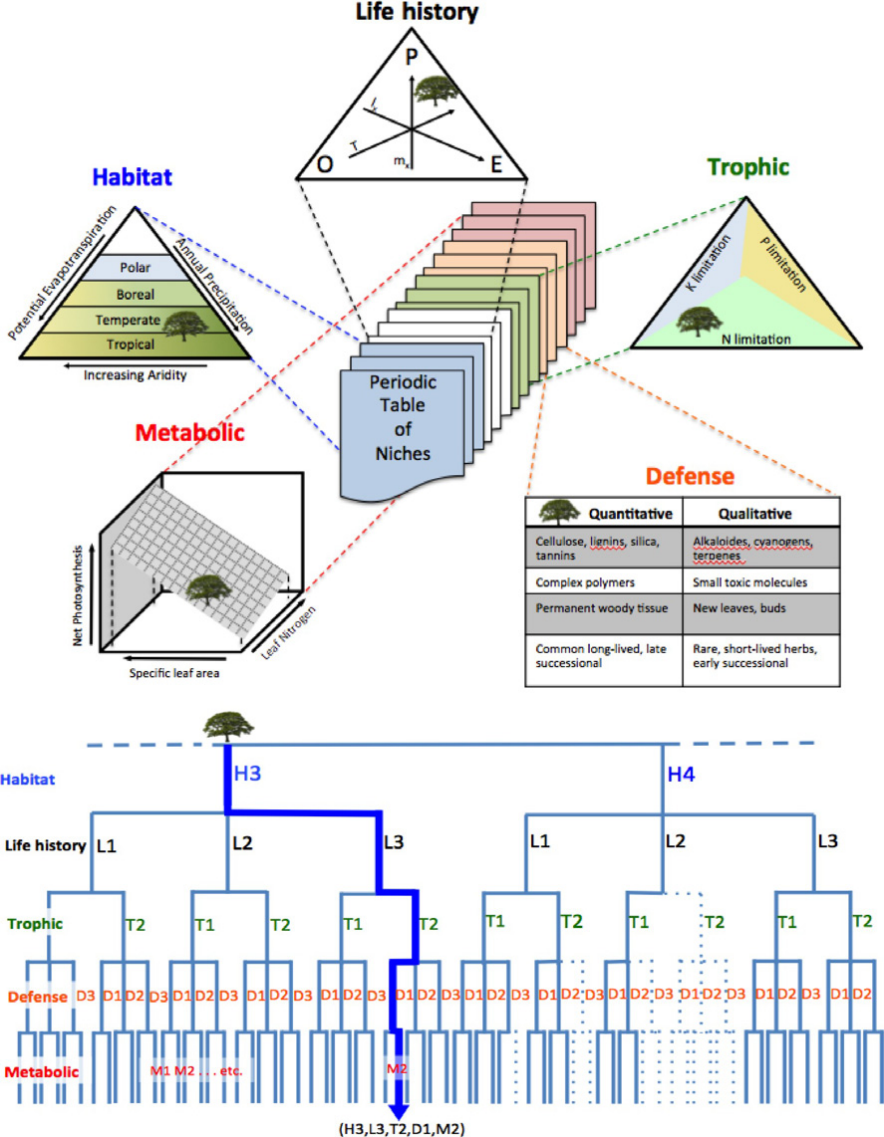
Illustration of the multidimensional nature of a periodic table of niches based on (a) relative position of a hypothetical tree species within ordination schemes for habitat, life history, trophic, defence and metabolic niche dimensions based on schemes adapted respectively from Holdridge (1967), Winemiller & Rose (1992), Wakefield *et al*. (2005), Feeny (1976), and Reich *et al*. (1997); and (b) a hypothetical classification tree, with the thick blue line representing a species entry for category H3,L3,T2,D1,M2 and dashed lines representing niche dimensional combinations unobserved in nature.

Obviously, the simplest form would be a two‐dimensional matrix similar to the periodic table of elements, with columns being trophic niches and rows representing life history strategies (Pianka 1974) or columns being modes of locomotion and rows representing some sort of evolutionary progression (McGhee 2011), and perhaps with separate tables for plants vs. animals or aquatic vs. terrestrial organisms. Such simple schemes might help introduce students to some basic ecological concepts, but would not be very useful for making predictions sufficiently specific for research applications. More helpful for ecologists and natural resource managers would be a scheme based on a limited number of fundamental niche dimensions (Table 1, Fig. 3) that discriminates niches using functional trait and performance data.

Any niche scheme assumes that certain species cluster around adaptive peaks defined by sets of trait combinations associated with a given set of environmental attributes within a given type of habitat and biome. Using multivariate methods of dimension‐reduction, the universe of possible trait combinations can be reduced to reveal sets of realised combinations corresponding to adaptive peaks in trait space (McGhee 2011; Verberk *et al*. 2013). Armed with this information, species could be clustered into a hierarchical niche scheme (Poff *et al*. 2010; Kleyer *et al*. 2012). The goal of a general niche scheme would be to determine the degree to which results from analysis of different data sets (involving different species and regions) are shown to be concordant. In practice, schemes derived in this manner would only pertain to certain higher taxa within certain subsets of habitat/ecosystem types. For example, relevant functional trait variation could not be captured in a table containing fungi and mammals because they are so different. Compared to the periodic table of elements, category entries within a periodic table of niches would be taxon dependent with imprecise boundaries. This, however, need not limit the scheme's utility, and ecologists already rely heavily on such methods. Our challenge is to gather and analyse more and better‐resolved data to sharpen blurred boundaries and improve predictions. Given the niche's multiple dimensions, a periodic table of niches might be viewed as a series of charts with a hierarchy of layers viewed in a hypertext format (Fig. 3). Here, we essentially are addressing the issue of information organisation for the end user; the more fundamental question is how best to derive a niche scheme?

Analyses aimed at producing a niche scheme should first divide the database of functional traits and performance measures into subsets based on their logical associations with each of five fundamental niche dimensions. Analyses then would be performed for each niche dimension separately, after which separate dimensional ordinations are integrated to produce interpretable niche schemes (Box 2 provides examples of two approaches). Analysis of data sets containing many functionally unrelated measures may fail to detect patterns of covariation that determine species’ ecological responses to and effects on their environments. Plant ecologists have sometimes attempted to differentiate plant functional types based on traits inferred to influence responses to environmental variation, and other traits based on effects the plant has on communities and ecosystems (Lavorel *et al*. 2007). Some functional traits might influence both responses and effects, but many others would not. Schemes are needed that organise observed trait combinations in a manner that allows us to specify how these relationships affect ecological responses and effects. Multivariate analysis of ecological responses and effects based on diverse collections of traits and performance measures, even when all have well‐documented functions, reveals many correlations (some functional but many spurious), but could mask important patterns associated with cause and effect. We need to think about niche dimensions *a priori* (based on the full weight of ecological knowledge) – not *a posteriori* (i.e. inferred from analysis of large data sets containing functionally unrelated variables).

Box 2 **Constructing a Niche Scheme for a Tropical Fish Assemblage**

We explored methods to create two alternative niche schemes: species ordination within continuous niche space vs. niche classification. Five data sets containing traits associated with five niche dimensions were compiled for a tropical freshwater fish assemblage of a floodplain creek in the Venezuelan Llanos (savanna region within the Orinoco River Basin) studied extensively by KOW. Data were obtained for 56 common fish species; an additional 33 species were collected in numbers insufficient to yield reliable data, and were excluded. Data were obtained for variables associated with five fundamental niche dimensions: habitat use, life history strategy, trophic ecology, defence, and metabolism/physiology. The five data sets (Tables S2‐7) collectively contained 38 variables pertaining to functional traits or ecological performance.

**Species ordination within separate niche dimensions**

PCA was performed on each dataset based on eigenanalysis of the correlation matrix using the VEGAN package in R 3.1.0 (Oksanen *et al*. 2013). Only the first two principal components (explaining between 38.6–74.8% of the variation for each niche dimension) were retained for further ordination and clustering (Tables S8–S12); criteria for the number of axes and amount of variation modelled for retention of dominant PC axes will depend, in part, on the scheme's intended applications.

**Species clustering based on trait similarities**

We performed a regression tree analysis on each niche dimension using the RPART package (Therneau *et al*. 2014). Species scores on the first two PCA components for each of the five data sets were used as response variables, and original traits were predictor variables. CART trees were then pruned using the 1‐SE rule to obtain final regression trees for each data set (Figs S15–S19). The number of terminal nodes of these trees (habitat = 4, life history = 5, trophic = 6, defence = 2, metabolic = 6) were used to define species groups. CART uses criteria based on values of the original variables for tree bifurcations, which facilitates interpretations.

**Construction of a continuous niche scheme**

A continuous niche scheme was created using PCA. Input data were species scores on the two dominant principal components from PCAs performed on each of the five data sets (Tables S8–S12). Ordination of species scores for this ‘PCA of PCAs’ (Table S13) represents a two‐dimensional continuum integrating patterns (strategies) within each of the five niche dimensions. Consequently, the five niche dimensions estimated by the five data sets have an equal chance to influence the overall niche gradients and species ordinations. Interpretation of these gradients is necessarily dependent upon interpretations of gradients obtained previously from PCAs of the five original data sets. The PCA of PCAs produced a dominant gradient that contrasted inactive, armoured, benthic fishes with surface‐oriented fishes that were active swimmers feeding on invertebrates, and a secondary gradient contrasting diverse microphagous species with opportunistic life history strategies and limited capacity for energy storage vs. large predators with equilibrium life history strategies.

**Construction of a niche classification**

For a niche classification scheme, dendrograms from CART analysis of the five data sets (Figs S14–S18) were combined in a hierarchy to construct a composite tree (Fig. 6, Figs S19–S22). Positions in the scheme represent combinations of trait values for each of five niche dimensions (e.g. *Hoplias malabaricus* (Bloch) – 2,4,2,1,3). The full dendrogram generated from this assemblage of 56 fishes has 1440 potential terminal nodes (the product of the number of groupings for each dimension). Only 50 of these potential nodes were occupied, with most potential trait combinations either non‐viable or vacant, and certain niches occupied by multiple species. For example, *Ctenobrycon spilurus* (Valenciennes) and *Pyrrhulina lugubris* Eigenmann occupied a common niche associated with omnivory, rapid and sustained movement during foraging and predator escape, high energy expenditure, limited fat storage and an opportunistic life history strategy. The number of terminal nodes depends on criteria chosen for clustering; nonetheless, this approach is useful for detection of vacant niches. Some trait combinations are unobserved because they are non‐viable (physically impossible or maladaptive), whereas others may be viable but not present with a given species assemblage or evolutionary lineage. The latter could occur for a host of reasons, including evolutionary or biogeographic contingencies (e.g. never evolved, or evolved but never dispersed into the region) or ecological factors (e.g. competitively inferior niches, niches incapable of persisting within a particular disturbance regime).

**Figure 4:**
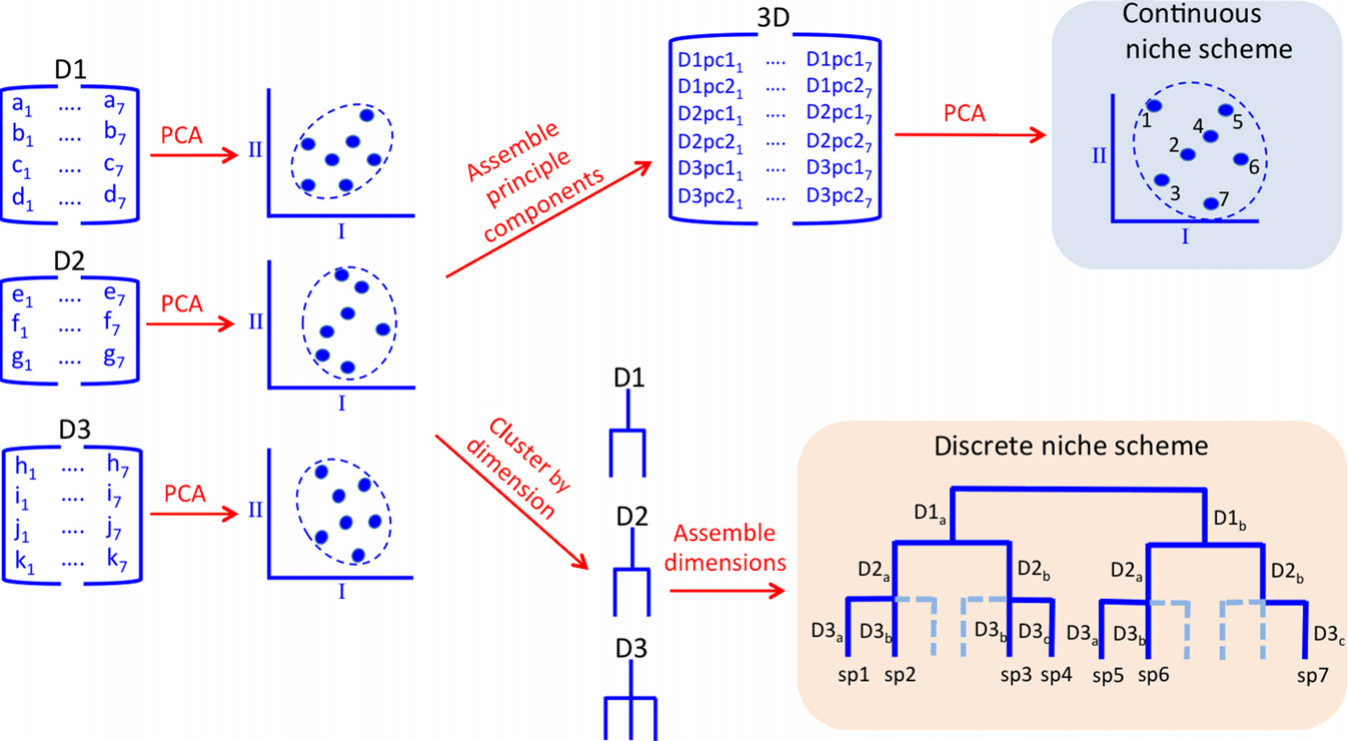
Schematic diagram for a general methodology for creating discrete and continuous niche schemes. D1, D2 and D3 are trait data matrices associated with three different niche dimensions involving a set of seven species. PCA axes I and II are dominant gradients of trait combinations derived from analyses performed on each data set separately. The continuous scheme derives from multivariate analysis using species loadings for the dominant axes from each dimensional analysis as input data. The discrete scheme is a niche classification derived from a cluster analysis, such as classification and regression tree, using interspecific distances based on species loadings from the continuous niche scheme and raw data for each niche dimension for classification. In practice, traits data would need to be standardised, data sets and steps in the process would need to be quality assured, and, in the case of the discrete scheme, clustering thresholds would need to be optimised for the intended use of the classification.

**Figure 5:**
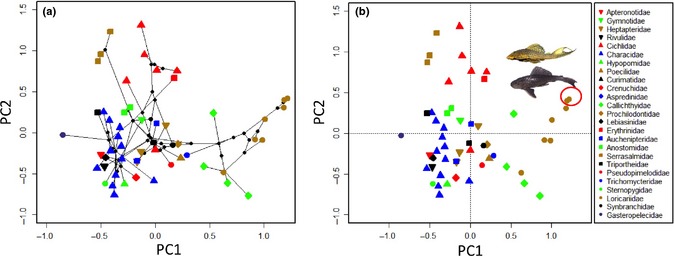
Example of a continuous niche scheme, a two‐dimensional ordination plot of tropical fish species based on analysis of 5‐dimensional niche space (i.e. PCA performed using species loadings on the two dominant axes from each of five separate PCAs). (a) Species plotted with symbols representing families, with network of lines representing phylogenetic relationships of species comprising the local assemblage and the length of each line representing the niche branch length between species or species and inferred ancestral nodes (method follows Sidlauskas 2008). (b) Species plotted with symbols as in (a) but without phylogenetic relationships and showing the location of two South American fishes that are invasive in the Southern U.S. and Mexico.

We explored two approaches to create a niche scheme using data for a diverse Neotropical fish assemblage: ordination within continuous gradients representing niche space, and categorisation of discrete niches based on clustering of species according to various niche dimensions. Details concerning the data set and analyses appear in Box 2. We compiled data sets containing traits associated with five niche dimensions: habitat use, life history strategy, trophic ecology, defence and metabolism/physiology. The first step in constructing a niche classification scheme is statistical data reduction to produce gradients of niche space with low dimensionality (Fig. 4). We first performed principal components analysis (PCA) on each of five niche dimensions separately. To create a continuous niche ordination, resulting component scores from the separate dimensional PCAs were then used in a second PCA. This created a continuous ordination of species relative positions within a two‐dimensional space that integrates the five niche dimensions based on dozens of functional traits and performance measures related in various ways to the different dimensions (Fig. 5).

**Figure 6:**
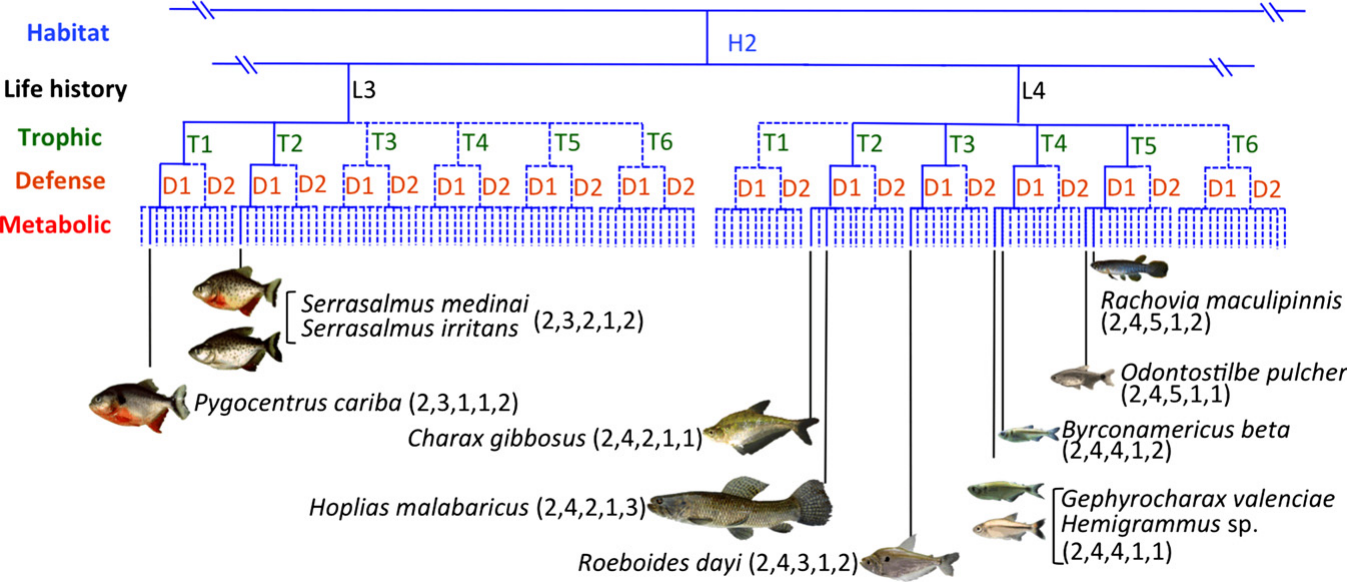
Example of a discrete niche classification scheme, dendrogram derived from classification and regression tree analysis. Species PC1 and PC2 scores were used as grouping criteria, and functional trait values associated with each niche dimension were the basis for bifurcations creating branching structure. Only a portion of the full dendrogram is shown here (remaining portions appear in Figs S19–S22). Each of two niches is occupied by two species, other niches are occupied by one species, and most potential trait combinations are unobserved in this diverse fish assemblage.

To create a discrete niche classification scheme, we first identified categories for each niche dimension independently. A variety of clustering algorithms and multivariate methods can be used to group organisms (e.g. k‐means clustering, UPGMA, classification and regression tree (CART) analysis), each of which will produce slightly different results. Criteria for grouping species affect the scheme's resolution; therefore, this approach must be guided by the intended applications of the classification and grounded in sound knowledge of the taxa. Quality assurance measures are required to ensure data are reliable with consistent scale and resolution to enable reasonable interspecific comparisons. Criteria for pruning regression trees and other methods are available to reduce subjectivity associated with clustering algorithms. As an example, we performed CART analysis using as response variables the species scores on the first two principal components derived from PCA of data sets compiled for each of five niche dimensions, using original trait and performance values as explanatory variables to create dendrograms (Fig. 4). Groupings obtained from the regression tree for five niche dimensions (Figs S14–S18) were then combined in a hierarchical manner (habitat → life history → trophic→ defence→ metabolic) to build a comprehensive niche classification (Fig. 6, Figs S19–S22). The particular hierarchical order in which niche dimensions are assembled determines positions of niche categories within the dendrogram, and therefore distances between categories are not interpretable. A logical hierarchy of organising the five fundamental niche dimensions might be – (1) habitat, (2) life history, (3) trophic, (4) defence and (5) metabolic (Table 1, Fig. 3).

Continuous niche schemes (Fig. 5) facilitate comparative research, such as investigation of adaptive divergence, niche conservatism and convergence, as well as studies of spatial or temporal variation in community structure and those aimed at predicting extinction vulnerability or invasion success. A discrete niche classification scheme, represented either as a dendrogram or table, is more directly analogous to chemistry's periodic table of elements. Discrete classification schemes would accommodate investigations of missing niches, models simulating dynamics of functional groups and transferability of biological assessments that rely on functional groups. We offer this example of a continuous vs. discrete approach with the aim of stimulating ecologists to collect more diverse kinds of traits data and to develop alternative methods for data analysis.

To explore the potential usefulness of our proposed approach, we compiled a data set containing mean abundance of each fish species at Caño Maraca based on 12 consecutive monthly surveys (Table S7) for comparison with species scores on the first two PC axes of each of five niche dimensions, and with species scores on first two axes from the PCA of the PCAs as described in Box 2. Pearson's correlations between mean abundance and each of the six pairs of dominant principal components were computed. Correlations also were computed between PC scores and the coefficient of variation (CV) of abundance, which served as a measure of population variability over the course of 1 year. This tropical stream drains a large floodplain and experiences a single, prolonged annual flood pulse that causes marked changes in the spatial extent of aquatic habitat, water quality (with periods of hypoxia), aquatic vegetation, food resource availability, fish density and diversity. We therefore expected that traits associated with all five niche dimensions could affect population dynamics, however it would be impossible to determine *a priori* if any single trait or dimension had a disproportionate influence. Highest correlation was between the coefficient of variation in abundance and PC1 from the PCA of PCAs (0.28), and the next highest correlation was between abundance and PC2 from the PCA of PCAs (0.18). By comparison, the same analysis performed using PC scores from the PCA performed using raw data for all 38 trait variables (Table S23) yielded lower correlations with the coefficient of variation in abundance (0.21 with PC2) and abundance (0.10 with PC1). Although much residual variation remained in both cases, the PCA of PCAs appears to describe relationships between niches and ecological responses more effectively.

Gradients derived from the PCA of PCAs seem to have more robust interpretations compared to those derived from the PCA performed using the data set of raw values for 38 traits (Tables S8–S12 and S23, and Fig. 5 and Fig. S24). The latter analysis yielded a dominant axis that described a gradient contrasting loricariid catfishes (wide body, armoured, long spines, large eggs, parental care, detritivory) with gymnotiform fishes and the synbranchid swamp eel (elongate body, unarmoured, no spines, small eggs, no parental care, invertivores). Because the 38‐trait data set was dominated by body shape variables compared to traits associated with other niche dimensions, body shape patterns dominated the gradients and species ordinations. The PCA of PCAs allows all five niche dimensions to have an equal opportunity to influence the composite niche scheme and species ordinations, with the gradients dominated by those dimensional components (themselves defined by combinations of traits and performance measures) having greatest influence on local community structure.

## Applications for Niche Classification Schemes

A number of important ecological applications already rely either explicitly or implicitly on niche schemes of one kind or another. Here, we mention three examples of applications that would benefit from transferable, robust niche schemes – invasion biology, biological indices for environmental assessment and restoration ecology. Other applications include assessment of species potential for biological control of pests based on functional traits (Snyder 2009) and simulation models that use functional groups with particular sets of functional traits to forecast ecosystem response to environmental stressors and global change (Steffen 1996; Elith & Leathwick 2009; Kearney *et al*. 2010).

A major goal in invasion biology is to identify sets of traits that may be used to forecast which species have greatest potential to be invasive (van Kleunen *et al*. 2010; Ordonez 2014). However, this approach generally has not allowed for the possibility that different combinations of traits, involving different niche dimensions, may be associated with invasion potential in a given environmental setting. Although invasive plants seem to share certain basic life history characteristics, we still cannot predict for specific locations why some species with these characteristics are successful invaders, even to the point of causing wholesale ecosystem change, while others fail to establish (Thompson & Davis 2011). The answer might depend upon interactions between two or more of the fundamental niche dimensions we have proposed. A niche ordination or classification based on combinations of functional trait and performance measures associated with separate niche dimensions could provide a more comprehensive assessment of a species’ potential to be invasive in a given community and habitat. A hypothetical example is a plant having trait combinations variously associated with habitat, life history, trophic and metabolic dimensions that confer high fitness in a given environment, actually realising a low fitness because it lacks suitable traits for defence against local herbivores. Evaluation of invasion potential based on a niche scheme that incorporates traits associated with key niche dimensions *a priori*, could improve prediction success by first screening in relation to a broad niche spectrum that then identifies niche dimensions and associated functional traits and performance measures to be examined in greater detail. The customary approach is to compile data sets containing as many traits as is feasible, and then attempt to identify the most influential traits associated with invasion success or failure in a particular scenario. Multivariate analyses based on this shotgun approach might fail to identify key sets of intercorrelated traits that define ecological strategies, because most of the correlations between these traits with other traits, including some that are spurious, weaken the pattern. In other words, the functional aspects (strategies) within a fundamental niche dimension could be obscured by inclusion of large numbers of ecologically relevant, correlated, yet functionally unrelated traits. For example, it is unlikely that life history strategies and their influence on population dynamics would be perceived in results from analysis of a large data set that combines just a few life history traits (e.g. propagule size, fecundity, age or size of maturation) with a much larger collection of traits associated with other niche dimensions.

Biological assessment frequently evaluates how the structure of impacted communities deviates from that of the pre‐impact natural community. Patrick (1949) pioneered the biological indicator approach using assemblage structure of benthic algae to assess degrees of impact from pollution. Indices of biotic integrity (IBIs) have since been developed for many taxonomic groups and are widely adopted by natural resource agencies and conservation organisations throughout the world. The basic assumption of IBIs is that degrees of change in distributions of functional groups reflect degrees of environmental degradation. These indices are computed from scores for component assemblage metrics involving coarse‐scale taxonomic and functional criteria (e.g. proportion of sample comprised of taxon X, proportions of sensitive vs. resistant species, etc.). Often we rely on reference communities from least‐impacted habitats, and these sometimes are not good matches for sites being assessed. In addition, spatial non‐transferability is a major limitation of IBIs. Ferraro (2013) argued that classifying habitats according to ecological periodic tables provides a means to improve ecological assessment. By more effectively identifying indicator taxa that predict how similar classes of disturbances affect certain niches irrespective of taxonomy, niche schemes might provide another objective means to assess impacts. If successful, this could greatly improve transferability of indices. The major advantage of IBIs for assessment is that methods are rapid and cheap; however, if practitioners could draw upon accepted niche schemes developed incrementally and tested repeatedly by the broader scientific community, increased precision and accuracy could be gained without sacrificing this advantage.

Some contend that restoration is a proving ground for ecological understanding (Young *et al*. 2005). Limiting similarity, ontogenetic niche shifts and species facilitation are but a few of the ecological concepts that directly influence approaches to restoration (Temperton *et al*. 2004). Important for successful restoration outcomes is determination of the extent to which communities assemble according to non‐random processes influenced by dispersal, environmental filtering and species interactions. Restoration ecology assumes that when native species are extirpated, important ecological functions are lost. Invasive species sometimes have niches formerly occupied by native species that had been extirpated (conserved or convergent niches), and depending on the degree of similarity, this may or may not inhibit reestablishment of lost native taxa. Efforts to restore native plant communities are increasingly adopting trait‐based approaches to deal with the challenge of forecasting responses to reintroduction practices (Gondard *et al*. 2003; Pywell *et al*. 2003; Laughlin 2014b). Could these efforts be enhanced by niche schemes that organise traits according to a hierarchy of niche dimensions, some more relevant to dispersal, others to defence and still others to resource acquisition? Interest has arisen for the idea of introducing functionally similar non‐native species into regions where major faunal elements have been lost due to human impacts. For example, a proposal for a Pleistocene rewilding of North America generated considerable controversy (Donlan *et al*. 2005). The argument that important ecological roles, such as shrub browsing or seed dispersal, are missing is grounded in the Eltonian niche concept. Apparently, no one has yet explored the degree of similarity required for claims of functional equivalence, nor has the problem been framed with respect to Hutchinson's *n*‐dimensional niche. A functionally equivalent seed disperser may or may not be equally capable of defence or demographic resilience to local patterns of environmental disturbances. A general niche scheme could enhance assessment of ecological restoration proposals.

## Conclusion

Some might argue that ecology has no analogy with the periodic table of elements and that any attempt to create a general niche scheme is bound to fail (Steffen 1996). We disagree and again note that considerable research within community ecology has focused on relationships between functional traits, ecological performance, functional groups and their potential to predict population, community and ecosystem responses to aspects of environmental variation. While a need to think about how to organise and visualise niche schemes remains, we propose that the initial step is to identify a limited and logical set of niche dimensions (we argue for five) and to collect robust and reliable trait and performance data related to each niche dimension.

Perhaps in the future, ecology will develop sufficient understanding of species niches and community structure to predict responses to environmental impacts and restoration efforts. The feasibility of niche schemes ultimately will depend on how we address some basic challenges. We need more rigorous criteria for identifying traits directly related to different niche dimensions (Blondel 2003; Bernhardt‐Römermann *et al*. 2008). Identification of functionally equivalent groups depends on the scale and resolution of traits as well as optimisation methods used to create a discrete niche classification scheme. Our discrete classification generated from an assemblage of 56 species resulted in 50 occupied niches (observed trait combinations) out of a possible 1440 niches (Box 2), suggesting that niche schemes constructed from larger data sets may become unwieldy. We anticipate that in the near future, computationally intensive algorithms for retrieval and analysis of massive trait and performance data matrices will be developed along lines analogous to those used to analyze massive amounts of genomics data obtained from next‐generation sequencing. Rigorous tests of predictive capabilities of niche schemes constructed using alternative methods and criteria must be developed. Niche schemes based on a consistent conceptual framework would enhance comparative research that analyses niche hypervolumes (Blonder *et al*. 2014) by facilitating comparisons among more diverse taxa.

Our goal here was to demonstrate the potential feasibility of a general niche scheme while exploring some conceptual and methodological issues. The ultimate test of such schemes will be their predictive capabilities and degree of utility in ecological applications. A universal periodic table of niches is unlikely, but instead alternative niche schemes could be developed for making predictions for different groups of organisms in different regions, or for addressing different kinds of problems.

In his 1972 chapter, Robert MacArthur offered the following insights:

But the science of ecology finally has some structure, even if not a very orderly structure as yet, and it is from the shortcomings of its present structure that we can make the safest predictions of the future.’ …. ‘Perhaps niche will turn out to be a concept that requires some subdivision into several precise definitions.

MacArthur's niche classification could be considered a metaphor for the direction ecology has taken over recent decades, and it might one day be possible to organize species according to an orderly structure. Actually, this is already happening – ecology is now strongly focused on functional traits, their patterns of constraint, and their relationship with environmental variables and community structure (Cadotte *et al*. 2013). Ecologists are creating schemes that ordinate species within niche dimensions to predict responses to environmental variation. Moreover, many ecological applications already assume we have sufficient knowledge about niches, when clearly many avenues remain to be explored.

## Authorship

KOW and ERP developed initial concepts; all authors reviewed the literature; and KOW, DBF and LMB compiled data, performed analyses and prepared figures, tables and appendices. KOW wrote the first draft of the manuscript, and all authors contributed substantially to revisions.

## References

[ele12462-bib-0001] Adler, P.B. , Stephen, P.E. & Jonathan, M.L. (2010). Coexistence of perennial plants: an embarrassment of niches. Ecol. Lett., 13, 1019–1029. 20545729 10.1111/j.1461-0248.2010.01496.x

[ele12462-bib-0002] Azzurro, E. , Tuset, V.M. , Lombarte, A. , Maynou, F. , Simberloff, D. , Rodríguez‐Pérez, A. *et al* (2014). External morphology explains the success of biological invasions. Ecol. Lett., 17, 1455–1463. 25227153 10.1111/ele.12351

[ele12462-bib-0003] Behmer, S.T. (2009). Insect herbivore nutrient regulation. Annu. Rev. Entomol., 54, 165–187. 18764740 10.1146/annurev.ento.54.110807.090537

[ele12462-bib-0004] Bernhardt‐Römermann, M. , Römermann, C. , Nuske, R. , Parth, A. , Klotz, S. , Schmidt, W. *et al* (2008). On the identification of the most suitable traits for plant functional trait analysis. Oikos, 117, 1533–1541.

[ele12462-bib-0005] Blondel, J. (2003). Guilds or functional groups: does it matter? Oikos, 100, 223–231.

[ele12462-bib-0006] Blonder, B. , Lamanna, C. , Violle, C. & Enquist, B.J. (2014). The *n*‐dimensional hypervolume. Global Ecol. Biogeogr., 23, 595–609.

[ele12462-bib-0007] Brusatte, S.L. , Benton, M.J. , Ruta, M. & Lloyd, G.T. (2008). Superiority, competition, and opportunism in the evolutionary radiation of dinosaurs. Science, 321, 1485–1488. 18787166 10.1126/science.1161833

[ele12462-bib-0008] Buckley, L.B. , Nufio, C.R. & Kingsolver, J.G. (2014). Phenotypic clines, energy balances and ecological responses to climate change. J. Anim. Ecol., 83, 41–50. 23662736 10.1111/1365-2656.12083

[ele12462-bib-0009] Cadotte, M. , Albert, C.H. & Walker, S.C. (2013). The ecology of differences: assessing community assembly with trait and evolutionary distances. Ecol. Lett., 16, 1234–1244. 23910526 10.1111/ele.12161

[ele12462-bib-0010] Colwell, R.K. & Rangel, R.T. (2009). Hutchinson's duality: the once and future niche. Proc. Natl. Acad. Sci. USA, 106, 19651–19658. 19805163 10.1073/pnas.0901650106 PMC2780946

[ele12462-bib-0011] Conway Morris, S. (2003). Life's Solution: Inevitable Humans in a Lonely Universe. Cambridge University Press, New York, NY.

[ele12462-bib-0012] Darling, E.S. , Alvarez‐Filip, L. , Oliver, T.A. , McClanahan, T.R. & Côté, I.M. (2012). Evaluating life‐history strategies of reef corals from species traits. Ecol. Lett., 15, 1378–1386. 22938190 10.1111/j.1461-0248.2012.01861.x

[ele12462-bib-0013] Donlan, J. , Greene, J. , Berger, C.E. , Bock, J.H. , Bock, D.A. , Burney, D.A. *et al* (2005). Re‐wilding North America. Nature, 436, 913–914. 16107817 10.1038/436913a

[ele12462-bib-0014] Dray, S. & Legendre, P. (2008). Testing the species traits‐environment relationships: the fourth‐corner problem revisited. Ecology, 89, 3400–3412. 19137946 10.1890/08-0349.1

[ele12462-bib-0015] Elith, J. & Leathwick, J.R. (2009). Species distribution models: ecological explanation and prediction across space and time. Annu. Rev. Ecol., Evol. Syst., 40, 677–697.

[ele12462-bib-0016] Emlen, D.J. (2008). The evolution of animal weapons. Annu. Rev. Ecol., Evol. Syst., 39, 387–413.

[ele12462-bib-0017] Feeny, P. (1976). Plant apparency and chemical defense In: Biochemical Interaction between Plants and Insects: Proceedings of the Fifteenth Annual Meeting of the Phytochemical Society of North America. (eds Wallace J.W. & Mansell R.L.). Plenum Press, New York, NY, pp. 1–40.

[ele12462-bib-0018] Ferraro, S.P. (2013). Ecological periodic tables: in principle and practice. Oikos, 122, 1541–1553.

[ele12462-bib-0019] Ferraro, S.P. & Cole, F.A. (2010). Ecological periodic tables for nekton usage of four US Pacific Northwest estuarine habitats. Can. J. Fish Aquat. Sci., 67, 1957–1967.

[ele12462-bib-0020] Frimpong, E.A. & Angermeier, P.L. (2009). Fish traits: a database of ecological and life‐history traits of freshwater fishes of the United States. Fisheries, 34, 487–495.

[ele12462-bib-0021] Golodets, C. , Sternberg, M. & Kigel, J. (2009). A community‐level test of the leaf‐height‐seed ecology strategy scheme in relation to grazing conditions. J. Veg. Sci., 20, 392–402.

[ele12462-bib-0022] Gondard, H. , Jauffret, S. , Aronson, J. & Lavorel, S. (2003). Plant functional types: a promising tool for management and restoration of degraded lands. Appl. Veg. Sci., 6, 223–234.

[ele12462-bib-0023] Grime, J.P. (1977). Evidence for existence of three primary strategies in plants and its relevance to ecological and evolutionary theory. Am. Nat., 111, 1169–1194.

[ele12462-bib-0024] Grime, J.P. (1979). Plant Strategies and Vegetation Processes. John Wiley & Sons, New York, NY.

[ele12462-bib-0025] Harmon, L.J. , Kolbe, J.J. , Cheverud, J.M. & Losos, J.B. (2005). Convergence and the multidimensional niche. Evolution, 59, 409–421. 15807425

[ele12462-bib-0026] Holdridge, L.R. (1967). Life Zone Ecology. Tropical Science Center, San Jose.

[ele12462-bib-0027] Holt, R.D. (2009). Bringing the Hutchinsonian niche into the 21st century: ecological and evolutionary perspectives. Proc. Natl. Acad. Sci. USA, 106, 19659–19665. 19903876 10.1073/pnas.0905137106 PMC2780934

[ele12462-bib-0028] Humphries, M.M. & McCann, K.S. (2014). Metabolic ecology. J. Anim. Ecol., 83, 7–19. 24028511 10.1111/1365-2656.12124

[ele12462-bib-0029] Ibañez, C. , Belliard, J. , Hughes, R.M. , Irz, P. , Kamdem‐Toham, A. & Lamouroux, N. (2009). Convergence of temperate and tropical stream fish assemblages. Ecography, 32, 658–670.

[ele12462-bib-0030] Inward, D.J. , Davies, R.G. , Pergande, C. , Denham, A.J. & Vogler, A.P. (2011). Local and regional ecological morphology of dung beetle assemblages across four biogeographic regions. J. Biogeogr., 38, 1668–1682.

[ele12462-bib-0031] Kattge, J. , Diaz, S. , Lavorel, S. , Prentice, I.C. , Leadley, P. , Bönisch, G. *et al* (2011). TRY–a global database of plant traits. Glob. Change Biol., 17, 2905–2935.

[ele12462-bib-0032] Kearney, M. , Simpson, S.J. , Raubenheimer, D. & Helmuth, B. (2010). Modelling the ecological niche from functional traits. Philos. Trans. R. Soc. B, 365, 3469–3483. 10.1098/rstb.2010.0034 PMC2981966 20921046

[ele12462-bib-0033] Keck, B.P. , Marion, Z.H. , Martin, D.J. , Kaufman, J.C. , Harden, C.P. , Schwartz, J.S. *et al* (2014). Fish functional traits correlated with environmental variables in a temperate biodiversity hotspot. PLoS ONE, 9, e93237. 24676053 10.1371/journal.pone.0093237 PMC3968117

[ele12462-bib-0034] van Kleunen, M. , Weber, E. & Fischer, M. (2010). A meta‐analysis of trait differences between invasive and non‐invasive plant species. Ecol. Lett., 13, 235–245. 20002494 10.1111/j.1461-0248.2009.01418.x

[ele12462-bib-0035] Kleyer, M. , Dray, S. , de Bello, F. , Leps, J. , Pakeman, R.J. , Strauss, B. *et al* (2012). Assessing species and community functional responses to environmental gradients: which multivariate methods? J. Veg. Sci., 23, 805–821.

[ele12462-bib-0036] Laughlin, D.C. (2014a). The intrinsic dimensionality of plant traits and its relevance to community assembly. J. Ecol., 102, 186–193.

[ele12462-bib-0037] Laughlin, D.C. (2014b). Applying trait‐based models to achieve functional targets for theory‐driven ecological restoration. Ecol. Lett., 17, 771–784. 24766299 10.1111/ele.12288

[ele12462-bib-0038] Laughlin, D.C. & Laughlin, D.E. (2013). Advances in modeling trait‐based plant community assembly. Trends Plant Sci., 18, 1360–1385. 10.1016/j.tplants.2013.04.012 23727200

[ele12462-bib-0039] Lavorel, S. , Díaz, S. , Cornelissen, J.H.C. , Garnier, E. , Harrison, S.P. , McIntyre, S. *et al* (2007). Plant functional types: are we getting any closer to the holy grail? In Terrestrial Ecosystems in a Changing World. (eds Canadell J.G., Pataki D., Pitelka L.). Springer‐Verlag, The IGBP Series, pp. 149–160.

[ele12462-bib-0040] MacArthur, R.H. (1972). Coexistence of species In: Challenging Biological Problems (ed Behnke J.A.). Oxford University Press, New York, NY, pp. 253–259.

[ele12462-bib-0041] Mahler, D.L. , Ingram, T. , Revell, L.J. & Losos, J.B. (2013). Exceptional convergence on the macroevolutionary landscape in island lizard radiations. Science, 341, 292–295. 23869019 10.1126/science.1232392

[ele12462-bib-0042] McGhee, G.R. (2011). Convergent Evolution: Limited Forms Most Beautiful. MIT Press, Cambridge, MA.

[ele12462-bib-0043] Mendeleev, D. (1869). Ueber die beziehungen der eigenschaften zu den atomgewichten der elemente. Zeitschrift für Chemie, 12, 405–406.

[ele12462-bib-0044] Mims, M.C. & Olden, J.D. (2012). Life history theory predicts fish assemblage response to hydrologic regimes. Ecology, 93, 35–45. 22486085 10.1890/11-0370.1

[ele12462-bib-0045] Miyazono, S. , Aycock, J.N. , Miranda, L.E. & Tietjen, T.E. (2010). Assemblage patterns of fish functional groups relative to habitat connectivity and conditions in floodplain lakes. Ecol. Freshw. Fish, 19, 578–585.

[ele12462-bib-0046] Moog, D. , Kahmen, S. & Poschlod, P. (2005). Application of CSR‐ and LHS‐strategies for the distinction of differently managed grasslands. Basic Appl. Ecol., 6, 133–143.

[ele12462-bib-0047] Mouillot, D. , Graham, N.A. , Villéger, S. , Mason, N.W.H. & Bellwood, D.R. (2013). A functional approach reveals community responses to disturbances. Trends Ecol. Evol., 28, 167–1677. 23141923 10.1016/j.tree.2012.10.004

[ele12462-bib-0048] Oksanen, J. , Blanchet, F.G. , Kindt, R. , Legendre, P. , Minchin, P.R. *et al* (2013). Vegan: community ecology package. R package version 2.0‐10. Available at: http://CRAN.R-project/package=vegan. Last accessed 15 May 2014.

[ele12462-bib-0049] Olden, J.D. , Poff, N.L. & Bestgen, K.R. (2006). Life history strategies predict fish invasions and extrirpations in the Colorado River Basin. Ecol. Monogr., 76, 25–40.

[ele12462-bib-0050] Ordonez, A. (2014). Functional and phylogenetic similarity of alien plants to co‐occurring natives. Ecology, 95, 1191–1202. 25000751 10.1890/13-1002.1

[ele12462-bib-0051] Patrick, R. (1949). A proposed biological measure of stream conditions based on a survey of Conestoga Basin, Lancaster County, Pennsylvania. Proc. Acad. Nat. Sci. Phila., 101, 277–341.

[ele12462-bib-0052] Pianka, E.R. (1974). Evolutionary Ecology. First Ed., Harper and Row, New York, NY.

[ele12462-bib-0053] Pianka, E.R. (1993). The many dimensions of a lizard's ecological niche In: Lacertids of the Mediterranean Basin (eds Valakos E.D., Bohme W., Perez‐Mellado V. & Maragou P.). Hellenic Zoological Society, University of Athens, Athens, Chapter 9, pp. 121–154.

[ele12462-bib-0054] Poff, N.L. , Pyne, M.I. , Bledsoe, B.P. , Cuhaciyan, C.C. & Carlisle, D.M. (2010). Developing linkages between species traits and multiscaled environmental variation to explore vulnerability of stream benthic communities to climate change. J. N. Am. Benthol. Soc., 29, 1441–1458.

[ele12462-bib-0055] Pywell, R.F. , Bullock, J.M. , Roy, D.B. , Warman, L.I.Z. , Walker, K.J. & Rothery, P. (2003). Plant traits as predictors of performance in ecological restoration. J. Appl. Ecol., 40, 65–77.

[ele12462-bib-0056] Reich, P.B. , Walters, M.B. & Ellsworth, D.S. (1997). From tropics to tundra: global convergence in plant functioning. Proc. Natl. Acad. Sci. USA, 94, 13730–13734. 9391094 10.1073/pnas.94.25.13730 PMC28374

[ele12462-bib-0057] Reich, P.B. , Tjoelker, M.G. , Machado, J.L. & Oleksyn, J. (2006). Universal scaling of respiratory metabolism, size and nitrogen in plants. Nature, 439, 457–461. 16437113 10.1038/nature04282

[ele12462-bib-0058] Rose, K.A. , Cowan, J.H. , Winemiller, K.O. , Myers, R.A. & Hilborn, R. (2001). Compensatory density dependence in fish populations: importance, controversy, understanding and prognosis. Fish Fish., 2, 293–327.

[ele12462-bib-0059] Sage, R.F. (2004). The evolution of C4 photosynthesis. New Phytol., 161, 341–370. 10.1111/j.1469-8137.2004.00974.x 33873498

[ele12462-bib-0060] Segar, S.T. , Pereira, R.A. , Compton, S.G. & Cook, J.M. (2013). Convergent structure of multitrophic communities over three continents. Ecol. Lett., 16, 1436–1445. 24134201 10.1111/ele.12183

[ele12462-bib-0061] Sidlauskas, B. (2008). Continuous and arrested morphological diversification in sister clades of characiform fishes: a phylomorphospace approach. Evolution, 62, 3135–3156. 18786183 10.1111/j.1558-5646.2008.00519.x

[ele12462-bib-0062] Smith, A.B. , Sandel, B. , Kraft, N.J. & Carey, S. (2013). Characterizing scale‐dependent community assembly using the functional‐diversity‐area relationship. Ecology, 94, 2392–2402. 24400491 10.1890/12-2109.1

[ele12462-bib-0063] Snyder, W.E. (2009). Coccinellids in diverse communities: which niche fits?. Biol. Control, 51, 323–335.

[ele12462-bib-0064] Soberón, J. & Nakamura, M. (2009). Niches and distributional areas: concepts, methods and assumptions. Proc. Natl. Acad. Sci. USA, 106, 19644–19650. 19805041 10.1073/pnas.0901637106 PMC2780935

[ele12462-bib-0065] Southwood, T.R.E. (1977). Habitat, the templet for ecological strategies? J. Anim. Ecol., 46, 337–365.

[ele12462-bib-0066] Steffen, W.L. (1996). A periodic table for ecology? A chemist's view of plant functional types. J. Veg. Sci., 7, 425–430.

[ele12462-bib-0067] Temperton, V.M. , Hobbs, R.J. , Nuttle, T. & Halle, S. , eds (2004). Assembly Rules and Restoration Ecology: Bridging the Gap Between Theory and Practice. Island Press, Washington, DC.

[ele12462-bib-0068] Therneau, T. , Atkinson, B. & Ripley, B. (2014). Rpart: recursive partitioning and regression trees. R package version 4.1‐8. Available at: http://CRAN.R-project.org/package=rpart. Last accessed: 15 May 2014.

[ele12462-bib-0069] Thompson, K. & Davis, M.A. (2011). Why research on traits of invasive plants tells us very little. Trends Ecol. Evol., 26, 155–156. 21334760 10.1016/j.tree.2011.01.007

[ele12462-bib-0070] Verberk, W.C.E.P. , Van Noordwijk, C.G.E. & Hildrew, A.G. (2013). Delivering on a promise: integrating species traits to transform descriptive community ecology into a predictive science. Freshw. Sci., 32, 531–547.

[ele12462-bib-0071] Wakefield, A.E. , Gotelli, N.J. , Wittman, S.E. & Ellison, A.M. (2005). Prey addition alters nutrient stoichiometry of the carnivorous plant *Sarracenia purpurea* . Ecology, 86, 1737–1743.

[ele12462-bib-0072] Westoby, M. (1998). A leaf–height–seed (LHS) plant ecology strategy scheme. Plant Soil, 199, 213–227.

[ele12462-bib-0073] Westoby, M. , Falster, D.S. , Moles, A.T. , Vesk, P.A. & Wright, I.J. (2002). Plant ecological strategies: some leading dimensions of variance between species. Annu. Rev. Ecol. Syst., 33, 125–159.

[ele12462-bib-0074] Wilson, J.B. & Lee, W.G. (2000). *C*‐*S*‐*R* triangle theory: community‐level predictions, tests, evaluation of criticisms, and relation to other theories. Oikos, 91, 77–96.

[ele12462-bib-0075] Winemiller, K.O. (1992). Life history strategies and the effectiveness of sexual selection. Oikos, 62, 318–327.

[ele12462-bib-0076] Winemiller, K.O. & Rose, K.A. (1992). Patterns of life‐history diversification in North American fishes: implications for population regulation. Can. J. Fish Aquat. Sci., 49, 2196–2218.

[ele12462-bib-0077] Young, T.P. , Petersen, D.A. & Clary, J.J. (2005). The ecology of restoration: historical links, emerging issues and unexplored realms. Ecol. Lett., 8, 662–673.

[ele12462-bib-0078] Zakon, H.H. (2002). Convergent evolution on the molecular level. Brain Behav. Evol., 59, 250–261. 12207082 10.1159/000063562

