# Supplementary material for: Functional traits, convergent evolution, and periodic tables of niches
Source: Ecol Lett. 2015 Jun 21;18(8):737–51. doi: 10.1111/ele.12462 (PMC4744997; doi:10.1111/ele.12462)
Supplement: Supplementary file 1 [file ELE-18-737-s001.docx]

**Supporting Information (Figs S14 - S**

Figure S14: Regression tree for the habitat dimension. Regression tree was constructed using the package RPART in R. Full tree was grown using the function rpart() with a minimum split condition of n=2 and subsequently pruned using the 1-SE criterion and the function prune(). The response variables used were PC1 and PC2 from the principal components analysis of the habitat dimension (Table S1) and the explanatory variables used were body depth, body width, body depth below midline (bdbm), mouth position, pectoral fin length, pectoral fin height, caudal fin length, caudal fin height, and pelvic fin length (pelv.l), with fin lengths expressed as ratios of standard length. Species move to the left when the stated condition is true. Species groupings at terminal nodes were subsequently used as the categories for each dimension while building the niche classification scheme in Box 2.

Figure S15: Regression tree for the life history dimension. Regression tree was constructed using the package RPART in R. Full tree was grown using the function rpart() with a minimum split condition of n=2 and subsequently pruned using the 1-SE criterion and the function prune(). Species move to the left when the stated condition is true. The response variables used were PC1 and PC2 from the principal components analysis of the life history dimension (Table S2) and the explanatory variables used were generation time, reproductive season (season), reproductive bouts, fecundity, egg diameter, parental care (parent), and standard length. Species groupings at terminal nodes were subsequently used as the categories for each dimension while building the niche classification scheme in Box 2.

Figure S16: Regression tree for the trophic dimension. Regression tree was constructed using the package RPART in R. Full tree was grown using the function rpart() with a minimum split condition of n=2 and subsequently pruned using the 1-SE criterion and the function prune(). Species move to the left when the stated condition is true. The response variables used were PC1 and PC2 from the principal components analysis of the trophic dimension (Table S3) and the explanatory variables used were volumetric proportions of detritus, algae, macrophytes, microorganisms, worms/molluscs, microcrustacea, decapod crustaceans (decapod), aquatic insects, terrestrial insects, and fish. Species groupings at terminal nodes were subsequently used as the categories for each dimension while building the niche classification scheme in Box 2.

Figure S17: Regression tree for the defense dimension. Regression tree was constructed using the package RPART in R. Full tree was grown using the function rpart() with a minimum split condition of n=2 and subsequently pruned using the 1-SE criterion and the function prune(). Species move to the left when the stated condition is true. The response variables used were PC1 and PC2 from the principal components analysis of the defense dimension (Table S4) and the explanatory variables used were spines, venom, armor, aggression, crypsis, speed, and body diameter. Species groupings at terminal nodes were subsequently used as the categories for each dimension while building the niche classification scheme in Box 2.

Figure S18: Regression tree for the metabolic dimension. Regression tree was constructed using the package RPART in R. Full tree was grown using the function rpart() with a minimum split condition of n=2 and subsequently pruned using the 1-SE criterion and the function prune(). Species move to the left when the stated condition is true. The response variables used were PC1 and PC2 from the principal components analysis of the metabolic dimension (Table S5) and the explanatory variables used were activity level (activity), hypoxia tolerance, visceral fat storage (fat), and special accessory respiration (respiration). Species groupings at terminal nodes were subsequently used as the categories for each dimension while building the niche classification scheme in Box 2.
