# Supplementary material for: Functional traits, convergent evolution, and periodic tables of niches
Source: Ecol Lett. 2015 Jun 21;18(8):737–51. doi: 10.1111/ele.12462 (PMC4744997; doi:10.1111/ele.12462)
Supplement: Supplementary file 2 [file ELE-18-737-s002.docx]

Figure S19: Additional segment of discrete PTN for Caño Maraca fish assemblage. Thick blue lines represent filled niches, dotted lines represent vacant niches for this assemblage. Description of categories for each niche dimension can be found in the text.

Figure S20: Additional segment of discrete PTN for Caño Maraca fish assemblage. Thick blue lines represent filled niches, dotted lines represent vacant niches for this assemblage. Description of categories for each niche dimension can be found in the text.

Figure S21: Additional segment of discrete PTN for Caño Maraca fish assemblage. Thick blue lines represent filled niches, dotted lines represent vacant niches for this assemblage. Description of categories for each niche dimension can be found in the text.

Figure S22: Additional segment of discrete PTN for Caño Maraca fish assemblage. Thick blue lines represent filled niches, dotted lines represent vacant niches for this assemblage. Description of categories for each niche dimension can be found in the text.
