# Supplementary material for: Functional traits, convergent evolution, and periodic tables of niches
Source: Ecol Lett. 2015 Jun 21;18(8):737–51. doi: 10.1111/ele.12462 (PMC4744997; doi:10.1111/ele.12462)
Supplement: Supplementary file 3 [file ELE-18-737-s003.docx]

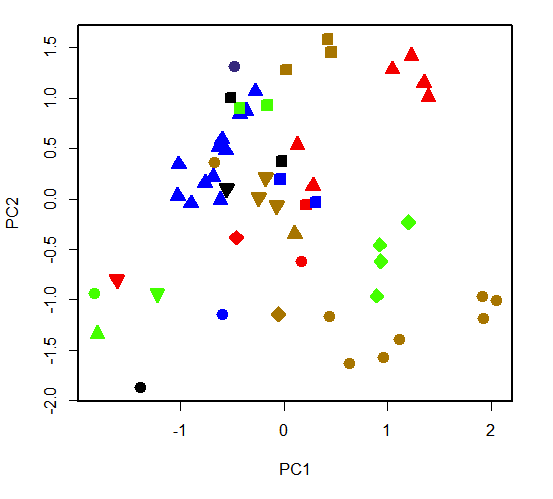


Figure S8: Principal components analysis of all 38 trait variables combined (i.e., not differentiated by niche dimension).
