# Supplementary material for: Functional traits, convergent evolution, and periodic tables of niches
Source: Ecol Lett. 2015 Jun 21;18(8):737–51. doi: 10.1111/ele.12462 (PMC4744997; doi:10.1111/ele.12462)
Supplement: Supplementary file 4 [file ELE-18-737-s004.docx]

| Species | body depth/sl | body width/sl | body dbml | head leng/sl | mouth position | rel pect leng | rel pect ht | rel caudal leng | rel caudal ht | rel pelvic leng | diel activity:  1diurnal, 2crepuscular,  3nocturnal | |
| --- | --- | --- | --- | --- | --- | --- | --- | --- | --- | --- | --- | --- |
| Adontosternarchus devananzii | 0.178 | 0.08 | 0.622 | 0.113 | 1 | 0.099 | 0.046 | 0.053 | 0.035 | 0 | 3 | |
| Aequidens pulcher | 0.49 | 0.249 | 0.507 | 0.353 | 2 | 0.376 | 0.168 | 0.314 | 0.319 | 0.366 | 1 | |
| Ancistrus sp. | 0.205 | 0.328 | 0.402 | 0.276 | 4 | 0.318 | 0.145 | 0.318 | 0.371 | 0.271 | 3 | |
| Aphyocharax alburnus | 0.28 | 0.137 | 0.475 | 0.261 | 2 | 0.199 | 0.063 | 0.243 | 0.256 | 0.159 | 1 | |
| Apistogramma hoignei | 0.393 | 0.19 | 0.446 | 0.337 | 2 | 0.263 | 0.137 | 0.303 | 0.219 | 0.274 | 1 | |
| Astronotus ocellatus | 0.518 | 0.227 | 0.475 | 0.361 | 1.3 | 0.287 | 0.2 | 0.258 | 0.358 | 0.24 | 1 | |
| Astyanax bimaculatus | 0.459 | 0.155 | 0.543 | 0.286 | 2 | 0.244 | 0.066 | 0.265 | 0.339 | 0.166 | 1 | |
| Brachyhypopomus sp. | 0.117 | 0.066 | 0.605 | 0.102 | 2 | 0.056 | 0.025 | 0 | 0 | 0 | 3 | |
| Bryconamericus beta | 0.362 | 0.142 | 0.502 | 0.247 | 2 | 0.219 | 0.092 | 0.29 | 0.358 | 0.153 | 1 | |
| Bunocephalus amaurus | 0.122 | 0.289 | 0.719 | 0.123 | 2 | 0.208 | 0.11 | 0.192 | 0.167 | 0.12 | 3 | |
| Caquetaia kraussii | 0.47 | 0.192 | 0.462 | 0.381 | 1 | 0.301 | 0.172 | 0.284 | 0.34 | 0.341 | 1 | |
| Characidium sp.1 | 0.287 | 0.138 | 0.484 | 0.254 | 3 | 0.254 | 0.087 | 0.274 | 0.208 | 0.225 | 1 | |
| Charax gibbosus | 0.378 | 0.124 | 0.527 | 0.253 | 1 | 0.185 | 0.055 | 0.25 | 0.3 | 0.204 | 1.5 | |
| Cheirodontops geayi | 0.309 | 0.109 | 0.427 | 0.219 | 1 | 0.211 | 0.06 | 0.279 | 0.253 | 0.125 | 1 | |
| Cichlasoma orinocense | 0.545 | 0.278 | 0.447 | 0.321 | 2 | 0.331 | 0.175 | 0.262 | 0.341 | 0.36 | 1 | |
| Corydoras aeneus | 0.408 | 0.302 | 0.301 | 0.277 | 4 | 0.26 | 0.109 | 0.364 | 0.393 | 0.208 | 1 | |
| Corydoras habrosus | 0.344 | 0.268 | 0.343 | 0.283 | 4 | 0.307 | 0.118 | 0.329 | 0.293 | 0.22 | 1 | |
| Corydoras septemtrionalis | 0.387 | 0.275 | 0.27 | 0.311 | 3 | 0.276 | 0.159 | 0.383 | 0.387 | 0.193 | 1 | |
| Crenicichla saxatilis | 0.214 | 0.177 | 0.541 | 0.318 | 1 | 0.21 | 0.124 | 0.196 | 0.229 | 0.179 | 1 | |
| Ctenobrycon spilurus | 0.457 | 0.134 | 0.514 | 0.236 | 2 | 0.229 | 0.069 | 0.26 | 0.284 | 0.165 | 1 | |
| Eigenmannia virescens | 0.156 | 0.074 | 0.541 | 0.109 | 2 | 0.083 | 0.036 | 0 | 0 | 0 | 3 | |
| Entomocorus gameroi | 0.282 | 0.214 | 0.448 | 0.252 | 1 | 0.265 | 0.131 | 0.257 | 0.362 | 0.177 | 3 | |
| Gephyrocharax valenciae | 0.277 | 0.113 | 0.658 | 0.243 | 1 | 0.208 | 0.056 | 0.242 | 0.254 | 0.136 | 1 | |
| Gymnotus carapo | 0.136 | 0.082 | 0.487 | 0.12 | 1 | 0.051 | 0.04 | 0 | 0 | 0 | 3 | |
| Hemigrammus sp. | 0.315 | 0.134 | 0.521 | 0.255 | 2 | 0.209 | 0.062 | 0.284 | 0.315 | 0.16 | 1 | |
| Hoplias malabaricus | 0.214 | 0.176 | 0.5 | 0.295 | 1 | 0.167 | 0.083 | 0.233 | 0.269 | 0.17 | 2 | |
| Hoplosternum littorale | 0.314 | 0.268 | 0.423 | 0.287 | 4 | 0.245 | 0.117 | 0.281 | 0.373 | 0.22 | 1.5 | |
| Hypoptopoma sp. | 0.206 | 0.267 | 0.398 | 0.281 | 4 | 0.309 | 0.117 | 0.323 | 0.258 | 0.133 | 1.5 | |
| Hypostomus argus | 0.22 | 0.327 | 0.362 | 0.185 | 4 | 0.334 | 0.189 | 0.42 | 0.427 | 0.268 | 3 | |
| Leporinus friderici | 0.337 | 0.182 | 0.468 | 0.246 | 3 | 0.184 | 0.138 | 0.224 | 0.364 | 0.182 | 1 | |
| Loricariichthys typus | 0.128 | 0.201 | 0.406 | 0.186 | 4 | 0.156 | 0.095 | 0.264 | 0.257 | 0.179 | 3 | |
| Markiana geayi | 0.48 | 0.165 | 0.594 | 0.282 | 3 | 0.225 | 0.098 | 0.228 | 0.325 | 0.142 | 1 | |
| Microglanis iheringi | 0.216 | 0.286 | 0.369 | 0.278 | 2 | 0.227 | 0.108 | 0.314 | 0.205 | 0.193 | 3 | |
| Ochmacanthus alternus | 0.154 | 0.175 | 0.293 | 0.165 | 4 | 0.134 | 0.066 | 0.125 | 0.117 | 0.084 | 3 | |
| Odontostilbe pulcher | 0.343 | 0.122 | 0.494 | 0.241 | 2 | 0.204 | 0.053 | 0.289 | 0.313 | 0.145 | 1 | |
| Otocinclus sp. | 0.218 | 0.234 | 0.37 | 0.27 | 4 | 0.226 | 0.067 | 0.264 | 0.253 | 0.164 | 1.5 | |
| Parauchenipterus galeatus | 0.283 | 0.283 | 0.484 | 0.27 | 1 | 0.219 | 0.104 | 0.245 | 0.262 | 0.144 | 3 | |
| Pimelodella sp2 | 0.195 | 0.183 | 0.462 | 0.23 | 3 | 0.181 | 0.109 | 0.245 | 0.243 | 0.164 | 3 | |
| Pimelodella sp3 | 0.186 | 0.175 | 0.458 | 0.236 | 3 | 0.185 | 0.096 | 0.401 | 0.196 | 0.165 | 3 | |
| Poecilia reticulata | 0.267 | 0.217 | 0.458 | 0.25 | 1 | 0.211 | 0.134 | 0.296 | 0.25 | 0.144 | 1 | |
| Prochilodus mariae | 0.414 | 0.185 | 0.436 | 0.288 | 3 | 0.224 | 0.072 | 0.214 | 0.39 | 0.21 | 1 | |
| Pterygoplichthys multirad. | 0.211 | 0.301 | 0.379 | 0.21 | 4 | 0.309 | 0.145 | 0.432 | 0.403 | 0.279 | 3 | |
| Pygocentrus cariba | 0.559 | 0.226 | 0.53 | 0.365 | 1 | 0.219 | 0.081 | 0.243 | 0.476 | 0.134 | 1.5 | |
| Pyrrhulina lugubris | 0.251 | 0.141 | 0.508 | 0.254 | 1 | 0.21 | 0.087 | 0.289 | 0.245 | 0.174 | 1 | |
| Rachovia maculipinnus | 0.285 | 0.207 | 0.58 | 0.296 | 1 | 0.254 | 0.128 | 0.345 | 0.22 | 0.148 | 1 | |
| Rhamdia sp. | 0.181 | 0.191 | 0.383 | 0.212 | 3 | 0.162 | 0.092 | 0.229 | 0.238 | 0.15 | 3 | |
| Rineloricaria caracasensis | 0.118 | 0.167 | 0.482 | 0.14 | 4 | 0.186 | 0.103 | 0.256 | 0.234 | 0.179 | 3 | |
| Roeboides dayi | 0.384 | 0.112 | 0.557 | 0.272 | 2 | 0.195 | 0.062 | 0.242 | 0.29 | 0.183 | 1 | |
| Schizodon isognathus | 0.291 | 0.174 | 0.456 | 0.253 | 2 | 0.169 | 0.079 | 0.216 | 0.354 | 0.187 | 1 | |
| Serrasalmus irritans | 0.546 | 0.157 | 0.518 | 0.308 | 1 | 0.22 | 0.09 | 0.175 | 0.428 | 0.117 | 1 | |
| Serrasalmus medinai | 0.596 | 0.181 | 0.544 | 0.357 | 1 | 0.226 | 0.212 | 0.29 | 0.499 | 0.15 | 1 | |
| Steindachnerina argentea | 0.388 | 0.188 | 0.459 | 0.297 | 3 | 0.204 | 0.062 | 0.303 | 0.382 | 0.235 | 1 | |
| Synbranchus marmoratus | 0.049 | 0.042 | 0.527 | 0.102 | 3 | 0 | 0 | 0 | 0 | 0 | 2.5 | |
| Tetragonopterus argenteus | 0.579 | 0.182 | 0.57 | 0.31 | 2 | 0.263 | 0.063 | 0.309 | 0.382 | 0.196 | 1 | |
| Thoracocharax stellatus | 0.58 | 0.153 | 0.852 | 0.273 | 1 | 0.491 | 0.149 | 0.318 | 0.37 | 0.054 | 1 | |
| Triportheus sp. | 0.367 | 0.135 | 0.652 | 0.241 | 1 | 0.368 | 0.093 | 0.152 | 0.288 | 0.135 | 1 | |
| See: Winemiller, K.O. 1991. Ecomorphological diversification of freshwater fish assemblages from five biotic regions. Ecological Monographs 61:343-365. | | | | | | | | | | | |  |
