# Supplementary material for: Functional traits, convergent evolution, and periodic tables of niches
Source: Ecol Lett. 2015 Jun 21;18(8):737–51. doi: 10.1111/ele.12462 (PMC4744997; doi:10.1111/ele.12462)
Supplement: Supplementary file 5 [file ELE-18-737-s005.docx]

| Species | gener time | reprod season | bouts | fecundity | egg dia | parent care | length |
| --- | --- | --- | --- | --- | --- | --- | --- |
|  | months | months | no/yr | batch |  | low-high | max standard |
| Adontosternarchus devananzii | 12 | 2 | 1 | 323 | 1.35 | 1 | 185 |
| Aequidens pulcher | 8 | 10 | 3 | 871 | 1.75 | 6 | 89 |
| Ancistrus sp. | 12 | 5 | 4 | 48 | 4 | 4 | 68 |
| Aphyocharax alburnus | 11 | 3 | 4 | 617 | 0.65 | 1 | 37 |
| Apistogramma hoignei | 4 | 12 | 2 | 59 | 0.65 | 4 | 30 |
| Astronotus ocellatus | 14 | 6 | 3 | 2301 | 2.4 | 7 | 181 |
| Astyanax bimaculatus | 12 | 2 | 1 | 4287 | 0.9 | 1 | 91 |
| Brachyhypopomus sp. | 12 | 2 | 3 | 323 | 0.75 | 1 | 184 |
| Bryconamericus beta | 4 | 12 | 6 | 796 | 0.095 | 1 | 44 |
| Bunocephalus amaurus | 12 | 3 | 4 | 178 | 1 | 1 | 58 |
| Caquetaia kraussii | 8 | 12 | 3 | 3702 | 1.85 | 6 | 230 |
| Characidium sp.1 | 11 | 4 | 2 | 154 | 0.65 | 1 | 28 |
| Charax gibbosus | 6 | 6 | 4 | 280 | 1 | 1 | 101 |
| Cheirodontops geayi | 6 | 2 | 1 | 1108 | 0.75 | 1 | 28 |
| Cichlasoma orinocense | 12 | 6 | 2 | 1287 | 1.85 | 6 | 112 |
| Corydoras aeneus | 12 | 2 | 1 | 152 | 1.45 | 2 | 44 |
| Corydoras habrosus | 12 | 4 | 3 | 12 | 0.7 | 2 | 25 |
| Corydoras septemtrionalis | 12 | 2 | 3 | 143 | 1.25 | 2 | 56 |
| Crenicichla saxatilis | 12 | 6 | 2 | 230 | 1.8 | 6 | 120 |
| Ctenobrycon spilurus | 11 | 3 | 3 | 755 | 0.75 | 1 | 52 |
| Eigenmannia virescens | 12 | 2 | 3 | 323 | 1.35 | 1 | 172 |
| Entomocorus gameroi | 12 | 2 | 2 | 238 | 0.85 | 1 | 42 |
| Gephyrocharax valenciae | 4 | 5 | 5 | 734 | 0.75 | 1 | 37 |
| Gymnotus carapo | 12 | 5 | 3 | 567 | 2 | 2 | 297 |
| Hemigrammus sp. | 4 | 8 | 4 | 282 | 0.75 | 1 | 33 |
| Hoplias malabaricus | 12 | 6 | 4 | 2462 | 2 | 2 | 352 |
| Hoplosternum littorale | 12 | 2 | 2 | 2684 | 2 | 4 | 218 |
| Hypoptopoma sp. | 12 | 2 | 4 | 52 | 1.6 | 1 | 60 |
| Hypostomus argus | 12 | 4 | 3 | 289 | 3.25 | 4 | 190 |
| Leporinus friderici | 12 | 2 | 1 | 100,000 | 1 | 1 | 252 |
| Loricariichthys typus | 12 | 4 | 5 | 421 | 3.05 | 4 | 224 |
| Markiana geayi | 12 | 2 | 1 | 3398 | 1 | 1 | 94 |
| Microglanis iheringi | 12 | 3 | 3 | 649 | 1.1 | 1 | 38 |
| Ochmacanthus alternus | 12 | 3 | 2 | 238 | 0.75 | 1 | 38 |
| Odontostilbe pulcher | 4 | 5 | 3 | 1108 | 0.75 | 1 | 36 |
| Otocinclus sp. | 8 | 4 | 4 | 52 | 0.9 | 1 | 26 |
| Parauchenipterus galeatus | 12 | 2 | 2 | 750 | 1.9 | 1 | 115 |
| Pimelodella sp2 | 12 | 2 | 2 | 4313 | 0.75 | 1 | 80 |
| Pimelodella sp3 | 12 | 2 | 2 | 1587 | 0.85 | 1 | 68 |
| Poecilia reticulata | 2 | 12 | 4 | 19 | 1.9 | 4 | 26 |
| Prochilodus mariae | 24 | 2 | 1 | 168348 | 1 | 1 | 260 |
| Pterygoplichthys multirad. | 12 | 3 | 3 | 763 | 3.5 | 4 | 233 |
| Pygocentrus cariba | 12 | 3 | 2 | 4303 | 2.25 | 2 | 248 |
| Pyrrhulina lugubris | 12 | 3 | 2 | 82 | 1 | 1 | 35 |
| Rachovia maculipinnus | 1 | 5 | 12 | 8 | 1.35 | 1 | 32 |
| Rhamdia sp. | 12 | 2 | 1 | 11585 | 1.05 | 1 | 200 |
| Rineloricaria caracasensis | 12 | 5 | 3 | 255 | 1.7 | 2 | 113 |
| Roeboides dayi | 3 | 12 | 6 | 280 | 1 | 1 | 72 |
| Schizodon isognathus | 12 | 2 | 1 | 28950 | 1 | 1 | 255 |
| Serrasalmus irritans | 12 | 3 | 3 | 3048 | 1.5 | 2 | 147 |
| Serrasalmus medinai | 12 | 3 | 3 | 3048 | 1.5 | 2 | 140 |
| Steindachnerina argentea | 12 | 3 | 2 | 3528 | 0.45 | 1 | 92 |
| Synbranchus marmoratus | 12 | 5 | 2 | 150 | 1.5 | 2 | 330 |
| Tetragonopterus argenteus | 12 | 2 | 1 | 3398 | 1 | 1 | 83 |
| Thoracocharax stellatus | 12 | 2 | 2 | 755 | 0.8 | 1 | 42 |
| Triportheus sp. | 12 | 2 | 1 | 3175 | 1.8 | 1 | 120 |
|  |  |  |  |  |  |  |  |
| See: Winemiller, K.O. 1989. Patterns of variation in life history among South American fishes in seasonal environments. Oecologia 81:225-241. | | | | | | | |
