# Supplementary material for: Functional traits, convergent evolution, and periodic tables of niches
Source: Ecol Lett. 2015 Jun 21;18(8):737–51. doi: 10.1111/ele.12462 (PMC4744997; doi:10.1111/ele.12462)
Supplement: Supplementary file 6 [file ELE-18-737-s006.docx]

| Species | detritus | algae | macro-  phytes | micro-  organisms | worms,  molluscs | micro  crustacea | decapod  crustac | aquatic  insects | terrestr  insects | fish |
| --- | --- | --- | --- | --- | --- | --- | --- | --- | --- | --- |
| Adontosternarchus devanan. | 0.0173 | 0 | 0.0076 | 0.0016 | 0.0026 | 0.2886 | 0 | 0.6481 | 0.0015 | 0 |
| Aequidens pulcher | 0.0903 | 0.007 | 0.3203 | 0 | 0.2748 | 0.0105 | 0.0074 | 0.2437 | 0.019 | 0.0072 |
| Ancistrus sp. | 0.9085 | 0.0431 | 0.002 | 0.0254 | 0 | 0 | 0 | 0 | 0 | 0 |
| Aphyocharax alburnus | 0.0019 | 0.0001 | 0.0013 | 0.0016 | 0 | 0.5729 | 0.0052 | 0.3385 | 0.0509 | 0 |
| Apistogramma hoignei | 0.0132 | 0.0162 | 0.1843 | 0.0129 | 0.0551 | 0.3864 | 0.003 | 0.3268 | 0.0002 | 0 |
| Astronotus ocellatus | 0.0086 | 0 | 0.0353 | 0.0001 | 0.079 | 0.0143 | 0.2831 | 0.0328 | 0.4617 | 0.1946 |
| Astyanax bimaculatus | 0.0059 | 0.1373 | 0.5104 | 0.0002 | 0 | 0.0233 | 0 | 0.1379 | 0.1449 | 0.0012 |
| Brachyhypopomus sp. | 0.0172 | 0 | 0.0034 | 0 | 0 | 0.3196 | 0 | 0.6564 | 0 | 0 |
| Bryconamericus beta | 0.0002 | 0.1069 | 0.4893 | 0.0022 | 0 | 0.0578 | 0.0034 | 0.2399 | 0.0966 | 0 |
| Bunocephalus amaurus | 0.0559 | 0.0046 | 0.1579 | 0.001 | 0.0009 | 0.0093 | 0.0913 | 0.564 | 0.0017 | 0 |
| Caquetaia kraussii | 0.0063 | 0.0005 | 0.0229 | 0.0004 | 0.0195 | 0.023 | 0.0555 | 0.2553 | 0.1468 | 0.4431 |
| Characidium sp.1 | 0.0055 | 0.0003 | 0.0055 | 0.0069 | 0.0041 | 0.1415 | 0 | 0.8226 | 0.0117 | 0 |
| Charax gibbosus | 0.0117 | 0 | 0.0063 | 0 | 0.0002 | 0.0029 | 0.0729 | 0.1768 | 0.0016 | 0.7474 |
| Cheirodontops geayi | 0.0061 | 0.0461 | 0.0307 | 0.106 | 0 | 0.7573 | 0 | 0.0307 | 0.0061 | 0 |
| Cichlasoma orinocense | 0.0519 | 0.0607 | 0.4756 | 0.0004 | 0.1811 | 0.0209 | 0.0017 | 0.1718 | 0.0021 | 0 |
| Corydoras aeneus | 0.0232 | 0.1649 | 0.0131 | 0.3664 | 0.002 | 0.1084 | 0 | 0.3204 | 0 | 0 |
| Corydoras habrosus | 0.0359 | 0.4737 | 0.017 | 0.3786 | 0 | 0.0184 | 0 | 0.0497 | 0 | 0 |
| Corydoras septentrionalis | 0.1479 | 0.0011 | 0.0014 | 0.0602 | 0 | 0.0197 | 0 | 0.7556 | 0 | 0.0005 |
| Crenicichla saxatilis | 0 | 0 | 0 | 0 | 0 | 0.0016 | 0 | 0.4389 | 0 | 0.5592 |
| Ctenobrycon spilurus | 0.0008 | 0.0133 | 0.5237 | 0.0035 | 0 | 0.0886 | 0.0004 | 0.0578 | 0.0003 | 0 |
| Eigenmannia virescens | 0.0037 | 0.0002 | 0.0031 | 0.0147 | 0.0003 | 0.5745 | 0 | 0.4023 | 0.0006 | 0 |
| Entomocorus gameroi | 0.003 | 0.0004 | 0.0035 | 0.3257 | 0 | 0.0256 | 0 | 0.2587 | 0.2868 | 0 |
| Gephyrocharax valenciae | 0.0017 | 0.0004 | 0.1591 | 0.0472 | 0.0056 | 0.1053 | 0.0002 | 0.2721 | 0.4042 | 0 |
| Gymnotus carapo | 0.005 | 0 | 0.0248 | 0 | 0.0479 | 0.0121 | 0.1581 | 0.3283 | 0.0295 | 0.3498 |
| Hemigrammus sp. | 0.0008 | 0.0742 | 0.0475 | 0.05 | 0 | 0.1841 | 0.0017 | 0.4964 | 0.1403 | 0 |
| Hoplias malabaricus | 0.0001 | 0 | 0.0121 | 0 | 0 | 0.0005 | 0.0018 | 0.0161 | 0.001 | 0.968 |
| Hoplosternum littorale | 0.2862 | 0.0006 | 0.0739 | 0.0136 | 0.1829 | 0.0147 | 0 | 0.3239 | 0.0379 | 0 |
| Hypoptopoma sp. | 0.654 | 0.2687 | 0.0025 | 0.0307 | 0 | 0.0017 | 0 | 0 | 0 | 0 |
| Hypostomus argus | 0.8216 | 0.0316 | 0.0025 | 0.0258 | 0 | 0 | 0 | 0.0002 | 0 | 0 |
| Leporinus friderici | 0.055 | 0.0001 | 0.4477 | 0.0465 | 0.0867 | 0.0011 | 0.02 | 0.0031 | 0 | 0.2912 |
| Loricariichthys typus | 0.5783 | 0.0199 | 0.0408 | 0.0697 | 0.0051 | 0.0391 | 0 | 0.1494 | 0 | 0 |
| Markiana geayi | 0.0297 | 0.0314 | 0.7625 | 0 | 0.0062 | 0.0056 | 0.0033 | 0.0486 | 0.0635 | 0 |
| Microglanis iheringi | 0.004 | 0 | 0.0083 | 0.0033 | 0 | 0.0314 | 0.0022 | 0.9357 | 0.0094 | 0 |
| Ochmacanthus alternus | 0.0526 | 0.0082 | 0.0004 | 0.0009 | 0 | 0 | 0 | 0.0003 | 0 | 0.9309 |
| Odontostilbe pulcher | 0.0194 | 0.6885 | 0.0257 | 0.0337 | 0 | 0.1728 | 0 | 0.0569 | 0 | 0 |
| Otocinclus sp. | 0.7753 | 0.1213 | 0.0012 | 0.0627 | 0 | 0.0008 | 0 | 0.0004 | 0 | 0 |
| Parauchenipterus galeatus | 0.005 | 0 | 0.0788 | 0.0009 | 0.0037 | 0.0014 | 0.0016 | 0.3513 | 0.4468 | 0.0511 |
| Pimelodella sp. 2 | 0.0525 | 0.0005 | 0.2164 | 0.0007 | 0.1819 | 0.0138 | 0.0071 | 0.1755 | 0.0231 | 0 |
| Pimelodella sp.3 | 0.017 | 0.0009 | 0.0255 | 0.0022 | 0.09 | 0.094 | 0.0726 | 0.4583 | 0.0044 | 0 |
| Poecilia reticulata | 0.3222 | 0.3372 | 0.0006 | 0.103 | 0 | 0.0359 | 0 | 0.0177 | 0.0007 | 0 |
| Prochilodus mariae | 0.812 | 0.0681 | 0.0048 | 0.0228 | 0 | 0 | 0 | 0.0112 | 0 | 0 |
| Pterygoplichthys multirad. | 0.7679 | 0.1054 | 0.0019 | 0.0156 | 0 | 0.0034 | 0 | 0.0006 | 0 | 0 |
| Pygocentrus cariba | 0.0365 | 0 | 0.0718 | 0 | 0 | 0.0004 | 0.238 | 0.0036 | 0.0121 | 0.6376 |
| Pyrrhulina lugubris | 0.0095 | 0.0574 | 0.0346 | 0.0088 | 0.0006 | 0.0153 | 0 | 0.2125 | 0.6569 | 0 |
| Rachovia maculipinnus | 0.0067 | 0.0016 | 0.0187 | 0.0019 | 0.027 | 0.1864 | 0 | 0.5884 | 0.1602 | 0 |
| Rhamdia sp. | 0.008 | 0 | 0.0561 | 0 | 0.0134 | 0.0251 | 0.0828 | 0.106 | 0.0832 | 0.4446 |
| Rineloricaria caracasensis | 0.7356 | 0.0703 | 0.0162 | 0.0397 | 0.0001 | 0.0176 | 0 | 0.049 | 0 | 0 |
| Roeboides dayi | 0.0009 | 0.0004 | 0.0036 | 0 | 0.0023 | 0.1381 | 0.0127 | 0.5565 | 0.0036 | 0.2553 |
| Schizodon isognathus | 0.0032 | 0.0063 | 0.9905 | 0 | 0 | 0 | 0 | 0 | 0 | 0 |
| Serrasalmus irritans | 0.0258 | 0.0001 | 0.029 | 0 | 0 | 0.0057 | 0 | 0.0089 | 0.0002 | 0.7856 |
| Serrasalmus medinai | 0.0004 | 0 | 0.0003 | 0 | 0 | 0.0394 | 0 | 0.1841 | 0 | 0.5292 |
| Steindachnerina argentea | 0.5763 | 0.1723 | 0.0266 | 0.0363 | 0 | 0.003 | 0 | 0 | 0 | 0 |
| Synbranchus marmoratus | 0.0177 | 0 | 0.006 | 0 | 0.0364 | 0.0508 | 0.0319 | 0.5644 | 0.0661 | 0.2236 |
| Tetragonopterus argenteus | 0.0219 | 0.0512 | 0.5542 | 0 | 0.0004 | 0.0007 | 0 | 0.2022 | 0.039 | 0.0134 |
| Thoracocharax stellatus | 0.0017 | 0.0001 | 0.0433 | 0.0003 | 0 | 0.0131 | 0 | 0.3449 | 0.5926 | 0 |
| Triportheus sp. | 0.0242 | 0.0003 | 0.6566 | 0.0012 | 0.0021 | 0.0086 | 0.0033 | 0.1055 | 0.1875 | 0 |
|  |  |  |  |  |  |  |  |  |  |  |
| See: Winemiller, K.O. and E.R. Pianka. 1990. Organization in natural assemblages of desert lizards and tropical fishes. Ecological Monographs 60:27-55. | | | | | | | | |  |  |
