# Supplementary material for: Functional traits, convergent evolution, and periodic tables of niches
Source: Ecol Lett. 2015 Jun 21;18(8):737–51. doi: 10.1111/ele.12462 (PMC4744997; doi:10.1111/ele.12462)
Supplement: Supplementary file 7 [file ELE-18-737-s007.docx]

|  | spines: | venom | armor | aggression | crypsis | speed | body diameter |
| --- | --- | --- | --- | --- | --- | --- | --- |
| Species | 0none,  1few small,  2few barbed,  3many thin,  4large | 0absent  1present | 0none,  1head only,  2body thin,  3body thick | 0none  1often w/o damage  2always damage | 0low,  1moderate  2extreme | 0slow  1moderate  2fast  3fast w/flight | In  dimension  w/  max.  measurement |
| Adontosternarchus devananzii | 0 | 0 | 0 | 0 | 2 | 1 | 25.3 |
| Aequidens pulcher | 3 | 0 | 0 | 1 | 1 | 1 | 36.1 |
| Ancistrus sp. | 4 | 0 | 3 | 0 | 1 | 0 | 26.3 |
| Aphyocharax alburnus | 0 | 0 | 0 | 0 | 0 | 2 | 10.2 |
| Apistogramma hoignei | 3 | 0 | 0 | 0 | 1 | 1 | 11.5 |
| Astronotus ocellatus | 3 | 0 | 0 | 1 | 1 | 1 | 98 |
| Astyanax bimaculatus | 0 | 0 | 0 | 0 | 0 | 2 | 25.8 |
| Brachyhypopomus sp.1 | 0 | 0 | 0 | 0 | 1 | 1 | 16.2 |
| Bryconamericus beta | 0 | 0 | 0 | 0 | 0 | 2 | 14.5 |
| Bunocephalus amaurus | 2 | 0 | 1 | 0 | 2 | 0 | 16.5 |
| Caquetaia kraussii | 3 | 0 | 0 | 1 | 1 | 1 | 69 |
| Characidium sp.1 | 0 | 0 | 0 | 0 | 1 | 1 | 8 |
| Charax gibbosus | 0 | 0 | 0 | 0 | 0 | 2 | 31.3 |
| Cheirodontops geayi | 0 | 0 | 0 | 0 | 0 | 2 | 8.2 |
| Cichlasoma orinocense | 3 | 0 | 0 | 1 | 1 | 1 | 52.3 |
| Corydoras aeneus | 1 | 1 | 2 | 0 | 0 | 1 | 14.8 |
| Corydoras habrosus | 1 | 1 | 2 | 0 | 0 | 1 | 6.7 |
| Corydoras septemtrionalis | 1 | 1 | 2 | 0 | 0 | 1 | 15.2 |
| Crenicichla saxatilis | 3 | 0 | 0 | 1 | 0 | 2 | 25.7 |
| Ctenobrycon spilurus | 0 | 0 | 0 | 0 | 0 | 2 | 18.7 |
| Eigenmannia virescens | 0 | 0 | 0 | 0 | 0 | 2 | 20.3 |
| Entomocorus gameroi | 2 | 0 | 0 | 0 | 0 | 1 | 10.5 |
| Gephyrocharax valenciae | 0 | 0 | 0 | 0 | 0 | 2 | 8.9 |
| Gymnotus carapo | 0 | 0 | 0 | 0 | 1 | 1 | 21.2 |
| Hemigrammus sp. | 0 | 0 | 0 | 0 | 0 | 2 | 10 |
| Hoplias malabaricus | 0 | 0 | 0 | 1 | 1 | 0 | 40 |
| Hoplosternum littorale | 3 | 0 | 3 | 0 | 0 | 0 | 38.6 |
| Hypoptopoma sp. | 4 | 0 | 3 | 0 | 1 | 0 | 12.8 |
| Hypostomus argus | 4 | 0 | 3 | 0 | 1 | 0 | 46.8 |
| Leporinus friderici | 0 | 0 | 0 | 0 | 0 | 2 | 32.9 |
| Loricariichthys typus | 1 | 0 | 3 | 0 | 2 | 0 | 40.6 |
| Markiana geayi | 0 | 0 | 0 | 0 | 0 | 2 | 32.2 |
| Microglanis iheringi | 2 | 0 | 0 | 0 | 1 | 0 | 8 |
| Ochmacanthus alternus | 0 | 0 | 0 | 0 | 0 | 0 | 6.2 |
| Odontostilbe pulcher | 0 | 0 | 0 | 0 | 0 | 2 | 9.9 |
| Otocinclus sp. | 1 | 0 | 2 | 0 | 0 | 0 | 6 |
| Parauchenipterus galeatus | 4 | 0 | 1 | 0 | 1 | 0 | 30.6 |
| Pimelodella sp. 2 | 2 | 1 | 0 | 0 | 0 | 2 | 14.6 |
| Pimelodella sp.3 | 2 | 1 | 0 | 0 | 0 | 2 | 12.2 |
| Poecilia reticulata | 0 | 0 | 0 | 0 | 0 | 1 | 5.8 |
| Prochilodus mariae | 0 | 0 | 0 | 0 | 0 | 2 | 47 |
| Pterygoplichthys multirad. | 4 | 0 | 3 | 0 | 1 | 0 | 44.7 |
| Pygocentrus cariba | 1 | 0 | 1 | 2 | 0 | 2 | 61.9 |
| Pyrrhulina lugubris | 0 | 0 | 0 | 0 | 0 | 2 | 9.4 |
| Rachovia maculipinnus | 0 | 0 | 0 | 0 | 0 | 2 | 8.5 |
| Rhamdia sp. | 2 | 1 | 0 | 0 | 0 | 1 | 41.4 |
| Rineloricaria caracasensis | 1 | 0 | 3 | 0 | 2 | 0 | 18.4 |
| Roeboides dayi | 0 | 0 | 0 | 0 | 0 | 2 | 20.6 |
| Schizodon isognathus | 0 | 0 | 0 | 0 | 0 | 2 | 45.2 |
| Serrasalmus irritans | 1 | 0 | 0 | 2 | 0 | 2 | 81.6 |
| Serrasalmus medinai | 1 | 0 | 0 | 2 | 0 | 2 | 60.8 |
| Steindachnerina argentea | 0 | 0 | 0 | 0 | 0 | 2 | 25.9 |
| Synbranchus marmoratus | 0 | 0 | 0 | 0 | 0 | 0 | 22 |
| Tetragonopterus argenteus | 0 | 0 | 0 | 0 | 0 | 2 | 28.8 |
| Thoracocharax stellatus | 0 | 0 | 0 | 0 | 0 | 3 | 20 |
| Triportheus sp. | 0 | 0 | 0 | 0 | 0 | 3 | 49.5 |
|  |  |  |  |  |  |  |  |
| spines: 0= none, 1= few short & weakly serrated, 2= few long or few short & strongly serrated, 3= many long (dorsal, anal), 4= long & massive   venom: 0= none, 1= pectoral & dorsal spines with venom  armor: 0= none, 1= massive skull, 2= thin bony plates covering body, 3= thick bony plates covering body  aggression: 0= none toward heterospecifics, 1= chasing/biting commonly observed toward heterospec., 2= chasing biting with cutting dentition that can cause major tissue damage  crypsis: 0= none, 1= mottling for blending with vegetation, 2= mimicry of vegetation of woody debris  speed: 0= slow and usually inactive, 1= slow and active, often good maneuverability, 2= fast and agile swimmer, 3= fast agile swimmer that also can escape by short aerial flight  body.diameter: maximum body dimension measured in cross section (measurement taken on largest specimen of the species) [note: the fish's girth and not its length sets the limit for ingestion by gape-limited predators] |  |  |  |  |  |  |  |
