# Supplementary material for: Functional traits, convergent evolution, and periodic tables of niches
Source: Ecol Lett. 2015 Jun 21;18(8):737–51. doi: 10.1111/ele.12462 (PMC4744997; doi:10.1111/ele.12462)
Supplement: Supplementary file 8 [file ELE-18-737-s008.docx]

| Species | activity level | hypoxia tolerance | | | visceral fat storage | | special accessory resp |  |
| --- | --- | --- | --- | --- | --- | --- | --- | --- |
|  | 1-sedentary, infreq bouts slow swimming,  2-slow swimmer at freq intervals,  3-slow continual swimmer, 4-constant swimming, frequent rapid bursts) | lowest DO value recorded among dates species was present at site | | | 1-lowest amount recorded,  4-highest amount recorded | | 0-none,  1-dermal lip protuberance,  2-accessory aerial respiration |  |
| Adontosternarchus devananzii | 3 | | 2.3 | 2.5 | | 0 | | |
| Aequidens pulcher | 3 | | 0.4 | 3.5 | | 0 | | |
| Ancistrus sp. | 2 | | 0.4 | 2 | | 2 | | |
| Aphyocharax alburnus | 4 | | 0.4 | 4 | | 1 | | |
| Apistogramma hoignei | 3 | | 0.4 | 2.5 | | 0 | | |
| Astronotus ocellatus | 3 | | 0.4 | 3 | | 0 | | |
| Astyanax bimaculatus | 4 | | 0.4 | 4 | | 1 | | |
| Brachyhypopomus sp.1 | 3 | | 0.4 | 3.5 | | 2 | | |
| Bryconamericus beta | 4 | | 1.5 | 3 | | 1 | | |
| Bunocephalus amaurus | 1 | | 0.4 | 3.5 | | 0 | | |
| Caquetaia kraussii | 3 | | 0.4 | 4 | | 0 | | |
| Characidium sp.1 | 2 | | 0.4 | 4 | | 0 | | |
| Charax gibbosus | 3 | | 1.5 | 3.5 | | 0 | | |
| Cheirodontops geayi | 4 | | 0.4 | 4 | | 1 | | |
| Cichlasoma orinocense | 3 | | 0.4 | 4 | | 2 | | |
| Corydoras aeneus | 2 | | 0.4 | 4 | | 2 | | |
| Corydoras habrosus | 2 | | 0.4 | 3 | | 2 | | |
| Corydoras septentrionalis | 2 | | 0.4 | 4 | | 2 | | |
| Crenicichla saxatilis | 3 | | 2.3 | 2 | | 0 | | |
| Ctenobrycon spilurus | 4 | | 0.4 | 3.5 | | 1 | | |
| Eigenmannia virescens | 3 | | 0.4 | 3.5 | | 0 | | |
| Entomocorus gameroi | 3 | | 1.5 | 3.5 | | 0 | | |
| Gephyrocharax valenciae | 4 | | 0.4 | 4 | | 1 | | |
| Gymnotus carapo | 3 | | 0.4 | 3 | | 2 | | |
| Hemigrammus sp. | 4 | | 1.5 | 3.5 | | 1 | | |
| Hoplias malabaricus | 1 | | 0.4 | 4 | | 1 | | |
| Hoplosternum littorale | 3 | | 0.4 | 4 | | 2 | | |
| Hypoptopoma sp. | 2 | | 1.5 | 1.5 | | 2 | | |
| Hypostomus argus | 2 | | 0.4 | 2 | | 2 | | |
| Leporinus friderici | 3 | | 2.3 | 3.5 | | 0 | | |
| Loricariichthys typus | 2 | | 0.4 | 2 | | 2 | | |
| Markiana geayi | 4 | | 0.4 | 4 | | 1 | | |
| Microglanis iheringi | 2 | | 0.4 | 4 | | 0 | | |
| Ochmacanthus alternus | 2 | | 0.4 | 2 | | 0 | | |
| Odontostilbe pulcher | 4 | | 0.4 | 3.5 | | 1 | | |
| Otocinclus sp. | 2 | | 0.4 | 3.5 | | 0 | | |
| Parauchenipterus galeatus | 3 | | 0.4 | 4 | | 0 | | |
| Pimelodella sp. 2 | 4 | | 0.4 | 4 | | 0 | | |
| Pimelodella sp.3 | 4 | | 0.4 | 4 | | 0 | | |
| Poecilia reticulata | 4 | | 0.4 | 2 | | 0 | | |
| Prochilodus mariae | 4 | | 0.4 | 3.5 | | 0 | | |
| Pterygoplichthys multirad. | 2 | | 0.4 | 2.5 | | 2 | | |
| Pygocentrus cariba | 3 | | 1.5 | 2.5 | | 0 | | |
| Pyrrhulina lugubris | 4 | | 0.4 | 3.5 | | 0 | | |
| Rachovia maculipinnus | 3 | | 2.3 | 2.5 | | 0 | | |
| Rhamdia sp. | 2 | | 0.4 | 3.5 | | 0 | | |
| Rineloricaria caracasensis | 2 | | 0.4 | 2.5 | | 2 | | |
| Roeboides dayi | 3 | | 0.4 | 3 | | 0 | | |
| Schizodon isognathus | 4 | | 1.5 | 2.5 | | 0 | | |
| Serrasalmus irritans | 3 | | 2.3 | 2 | | 0 | | |
| Serrasalmus medinai | 3 | | 2.3 | 2.5 | | 0 | | |
| Steindachnerina argentea | 4 | | 0.4 | 3 | | 0 | | |
| Synbranchus marmoratus | 1 | | 1.5 | 1 | | 2 | | |
| Tetragonopterus argenteus | 4 | | 2.3 | 2.5 | | 1 | | |
| Thoracocharax stellatus | 4 | | 0.4 | 4 | | 1 | | |
