# Supplementary material for: Functional traits, convergent evolution, and periodic tables of niches
Source: Ecol Lett. 2015 Jun 21;18(8):737–51. doi: 10.1111/ele.12462 (PMC4744997; doi:10.1111/ele.12462)
Supplement: Supplementary file 9 [file ELE-18-737-s009.docx]

| Species | Mar-84 | Apr-84 | May-84 | Jun-84 | Jul-84 | Aug-84 | Sep-84 | Oct-84 | Nov-84 | Dec-84 |
| --- | --- | --- | --- | --- | --- | --- | --- | --- | --- | --- |
| Adontosternarchus devanan. | 0 | 0 | 0 | 0 | 0 | 10 | 25 | 0 | 0 | 23 |
| Aequidens pulcher | 58 | 16 | 10 | 10 | 91 | 89 | 117 | 99 | 94 | 39 |
| Ancistrus sp. | 4 | 42 | 2 | 0 | 0 | 0 | 0 | 0 | 0 | 3 |
| Aphyocharax alburnus | 19 | 10 | 3 | 11 | 68 | 65 | 244 | 108 | 63 | 46 |
| Apistogramma hoignei | 38 | 17 | 9 | 0 | 1 | 5 | 6 | 7 | 35 | 12 |
| Astronotus ocellatus | 4 | 16 | 1 | 2 | 3 | 90 | 1 | 3 | 20 | 16 |
| Astyanax bimaculatus | 4 | 7 | 1 | 207 | 306 | 73 | 300 | 90 | 95 | 147 |
| Brachyhypopomus sp.1 | 5 | 4 | 1 | 1 | 0 | 2 | 7 | 5 | 16 | 17 |
| Bryconamericus beta | 0 | 0 | 0 | 1 | 21 | 16 | 6 | 3 | 1 | 3 |
| Bunocephalus amaurus | 82 | 195 | 129 | 5 | 7 | 14 | 89 | 131 | 90 | 49 |
| Caquetaia kraussii | 14 | 38 | 4 | 10 | 254 | 69 | 90 | 95 | 100 | 63 |
| Characidium sp.1 | 3 | 6 | 5 | 1 | 0 | 12 | 56 | 69 | 30 | 35 |
| Charax gibbosus | 0 | 0 | 0 | 7 | 39 | 29 | 54 | 79 | 50 | 65 |
| Cheirodontops geayi | 24 | 13 | 0 | 0 | 0 | 0 | 1 | 7 | 2 | 15 |
| Cichlasoma orinocense | 30 | 37 | 11 | 87 | 20 | 60 | 19 | 16 | 24 | 20 |
| Corydoras aeneus | 24 | 205 | 24 | 2 | 1 | 0 | 0 | 29 | 62 | 3 |
| Corydoras habrosus | 102 | 164 | 192 | 0 | 0 | 0 | 11 | 66 | 17 | 87 |
| Corydoras septemtrionalis | 34 | 62 | 11 | 0 | 3 | 3 | 11 | 10 | 13 | 49 |
| Crenicichla saxatilis | 0 | 0 | 0 | 0 | 0 | 2 | 1 | 0 | 9 | 4 |
| Ctenobrycon spilurus | 200 | 196 | 5 | 212 | 250 | 115 | 409 | 387 | 167 | 185 |
| Eigenmannia virescens | 3 | 8 | 9 | 1 | 2 | 12 | 28 | 5 | 130 | 67 |
| Entomocorus gameroi | 0 | 0 | 0 | 0 | 2 | 5 | 25 | 7 | 1 | 15 |
| Gephyrocharax valenciae | 18 | 0 | 0 | 97 | 240 | 71 | 189 | 258 | 159 | 135 |
| Gymnotus carapo | 6 | 35 | 23 | 6 | 28 | 30 | 51 | 63 | 60 | 49 |
| Hemigrammus sp. | 1 | 0 | 0 | 4 | 2 | 43 | 70 | 71 | 8 | 29 |
| Hoplias malabaricus | 20 | 29 | 0 | 95 | 73 | 76 | 46 | 25 | 87 | 27 |
| Hoplosternum littorale | 47 | 50 | 64 | 7 | 6 | 3 | 0 | 2 | 9 | 23 |
| Hypoptopoma sp. | 3 | 0 | 0 | 0 | 8 | 15 | 6 | 4 | 1 | 4 |
| Hypostomus argus | 40 | 42 | 45 | 47 | 12 | 38 | 9 | 40 | 43 | 22 |
| Leporinus friderici | 0 | 0 | 0 | 1 | 0 | 3 | 24 | 0 | 24 | 17 |
| Loricariichthys typus (=platymetopon) | 36 | 44 | 40 | 61 | 58 | 37 | 84 | 76 | 39 | 19 |
| Markiana geayi | 6 | 4 | 0 | 170 | 178 | 52 | 238 | 35 | 191 | 137 |
| Microglanis iheringi | 17 | 82 | 24 | 3 | 18 | 50 | 43 | 16 | 11 | 11 |
| Ochmacanthus alternus | 72 | 14 | 8 | 1 | 30 | 26 | 115 | 107 | 28 | 63 |
| Odontostilbe pulcher | 200 | 159 | 73 | 206 | 352 | 72 | 182 | 900 | 173 | 310 |
| Otocinclus sp. | 80 | 144 | 193 | 2 | 3 | 5 | 11 | 12 | 15 | 69 |
| Parauchenipterus galeatus | 5 | 36 | 5 | 8 | 13 | 9 | 39 | 4 | 53 | 29 |
| Pimelodella sp. 2 | 9 | 9 | 1 | 0 | 9 | 1 | 138 | 14 | 79 | 66 |
| Pimelodella sp.3 | 19 | 21 | 40 | 0 | 23 | 5 | 13 | 15 | 22 | 35 |
| Poecilia reticulata | 88 | 1 | 0 | 16 | 75 | 20 | 26 | 55 | 2 | 14 |
| Prochilodus mariae | 36 | 36 | 42 | 41 | 87 | 28 | 52 | 76 | 42 | 85 |
| Pterygoplichthys multirad. | 36 | 41 | 47 | 138 | 52 | 33 | 4 | 46 | 37 | 6 |
| Pygocentrus cariba | 0 | 0 | 0 | 14 | 34 | 17 | 46 | 0 | 68 | 59 |
| Pyrrhulina lugubris | 17 | 2 | 0 | 2 | 1 | 14 | 1 | 58 | 123 | 98 |
| Rachovia maculipinnus | 0 | 0 | 0 | 24 | 74 | 25 | 7 | 0 | 1 | 0 |
| Rhamdia sp. | 2 | 30 | 2 | 20 | 13 | 4 | 18 | 7 | 10 | 9 |
| Rineloricaria caracasensis | 63 | 48 | 79 | 0 | 72 | 75 | 56 | 47 | 7 | 48 |
| Roeboides dayi | 7 | 7 | 12 | 72 | 181 | 171 | 169 | 251 | 113 | 102 |
| Schizodon isognathus | 0 | 0 | 0 | 3 | 8 | 3 | 33 | 1 | 33 | 11 |
| Serrasalmus irritans | 0 | 0 | 0 | 4 | 21 | 26 | 24 | 0 | 0 | 1 |
| Serrasalmus medinai | 0 | 0 | 0 | 0 | 15 | 39 | 3 | 0 | 6 | 4 |
| Steindachnerina argentea | 23 | 136 | 12 | 263 | 216 | 163 | 353 | 261 | 170 | 630 |
| Synbranchus marmoratus | 2 | 0 | 3 | 3 | 15 | 16 | 26 | 17 | 8 | 3 |
| Tetragonopterus argenteus | 0 | 0 | 0 | 0 | 9 | 0 | 52 | 25 | 83 | 31 |
| Thoracocharax stellatus | 82 | 6 | 0 | 0 | 2 | 19 | 27 | 18 | 56 | 86 |
| Triportheus sp. | 9 | 2 | 0 | 25 | 141 | 37 | 37 | 60 | 180 | 233 |

See: Winemiller, K.O. 1996. Dynamic diversity: Fish communities of tropical rivers. Pages 99-134, In: M.L. Cody and J.A. Smallwood, eds. Long-term Studies of Vertebrate Communities. Academic Press, Orlando, Florida.

Note: species were included in the dataset when they occurred in 4 or more months and samples included both adults and juveniles; plus Triportheus angulatus was excluded because life history data were incomplete.
