# Supplementary material for: Functional traits, convergent evolution, and periodic tables of niches
Source: Ecol Lett. 2015 Jun 21;18(8):737–51. doi: 10.1111/ele.12462 (PMC4744997; doi:10.1111/ele.12462)
Supplement: Supplementary file 10 [file ELE-18-737-s010.docx]

**Supplemental Information**

Table S8: The eigenvalues, proportion of variance explained, eigenvectors (variable scores), and species scores from principal component analysis yielding species ordination of the ecomorphological data relating to habitat use. Species scores are weighted by sums of species scores.

|  | PC1 | PC2 | PC3 | PC4 | PC5 | PC6 |
| --- | --- | --- | --- | --- | --- | --- |
| Eigenvalue | 5.368 | 2.118 | 0.723 | 0.533 | 0.338 | 0.299 |
| Proportion Explained | 0.537 | 0.212 | 0.072 | 0.053 | 0.034 | 0.030 |
| Cumulative Proportion | 0.537 | 0.749 | 0.821 | 0.874 | 0.908 | 0.938 |
| Variable Scores | PC1 | PC2 | PC3 | PC4 | PC5 | PC6 |
| Relative body depth | 0.969 | -0.953 | 0.484 | -0.113 | 0.403 | -0.055 |
| Relative body width | 1.167 | 0.670 | -0.443 | 0.137 | 0.169 | 0.236 |
| Relative body depth below midline | -0.488 | -1.186 | -0.517 | -0.542 | -0.080 | -0.168 |
| Relative head length | 1.255 | -0.536 | 0.411 | 0.319 | -0.035 | -0.074 |
| Mouth position | 0.210 | 1.284 | 0.254 | -0.642 | 0.298 | -0.228 |
| Relative pectoral length | 1.305 | -0.281 | -0.413 | -0.384 | 0.004 | -0.060 |
| Relative pectoral fin height | 1.217 | 0.032 | -0.683 | 0.344 | 0.310 | -0.122 |
| Relative caudal fin length | 1.324 | 0.234 | -0.015 | -0.265 | -0.536 | 0.288 |
| Relative caudal fin height | 1.334 | -0.262 | 0.327 | -0.239 | 0.091 | 0.336 |
| Relative pelvic fin length | 1.309 | 0.255 | 0.138 | 0.131 | -0.335 | -0.586 |
| Species Scores | PC1 | PC2 | PC3 | PC4 | PC5 | PC6 |
| *Adontosternarchus devananzii* | -1.472 | -0.443 | -0.670 | 0.206 | 0.278 | 0.125 |
| *Aequidens pulcher* | 0.981 | -0.298 | -0.408 | 0.168 | -0.047 | -1.863 |
| *Ancistrus sp.* | 0.710 | 0.938 | -0.626 | -0.223 | 0.125 | -0.236 |
| *Aphyocharax alburnus* | -0.205 | -0.054 | 0.541 | -0.077 | -0.547 | -0.055 |
| *Apistogramma hoignei* | 0.456 | -0.064 | 0.098 | 0.630 | -0.344 | -1.302 |
| *Astronotus ocellatus* | 0.768 | -0.558 | -0.344 | 1.072 | 0.966 | -0.605 |
| *Astyanax bimaculatus* | 0.093 | -0.504 | 0.683 | -0.674 | -0.015 | 0.024 |
| *Brachyhypopomus sp.* | -1.740 | 0.000 | -0.296 | -0.097 | 0.625 | -0.530 |
| *Bryconamericus beta* | 0.035 | -0.234 | 0.382 | -0.445 | -0.172 | 0.391 |
| *Bunocephalus amaurus* | -0.469 | 0.156 | -2.218 | -0.770 | 0.032 | 0.087 |
| *Caquetaia kraussii* | 0.813 | -0.603 | 0.000 | 1.256 | -0.339 | -1.437 |
| *Characidium sp.* | -0.005 | 0.183 | 0.223 | -0.523 | -0.586 | -1.226 |
| *Charax gibbosus* | -0.153 | -0.604 | 0.669 | 0.159 | -1.029 | -0.120 |
| *Cheirodontops geayi* | -0.291 | -0.284 | 0.491 | 0.330 | -0.978 | 0.832 |
| *Cichlasoma orinocense* | 0.947 | -0.128 | -0.240 | 0.589 | 0.733 | -1.619 |
| *Corydoras aeneus* | 0.677 | 0.876 | 0.675 | -0.122 | 0.648 | 0.857 |
| *Corydoras habrosus* | 0.564 | 0.782 | 0.195 | -0.193 | 0.422 | -0.093 |
| *Corydoras septemtrionalis* | 0.801 | 0.721 | 0.347 | 0.649 | 0.654 | 0.983 |
| *Crenicichla saxatilis* | -0.055 | -0.413 | -0.544 | 1.061 | -0.484 | -0.438 |
| *Ctenobrycon spilurus* | -0.063 | -0.370 | 0.605 | -0.599 | -0.015 | -0.158 |
| *Eigenmannia virescens* | -1.603 | 0.066 | -0.224 | 0.134 | 0.979 | -0.459 |
| *Entomocorus gameroi* | 0.238 | -0.187 | -0.500 | 0.734 | -0.190 | 0.728 |
| *Gephyrocharax valenciae* | -0.390 | -0.805 | -0.119 | -0.437 | -1.173 | 0.064 |
| *Gymnotus carapo* | -1.623 | -0.053 | -0.205 | 1.196 | 0.565 | 0.103 |
| *Hemigrammus sp.* | -0.110 | -0.221 | 0.538 | -0.544 | -0.698 | 0.190 |
| *Hoplias malabaricus* | -0.153 | -0.267 | 0.057 | 0.908 | -0.963 | 0.300 |
| *Hoplosternum littorale* | 0.462 | 0.600 | 0.199 | -0.338 | 0.592 | -0.103 |
| *Hypoptopoma sp.* | 0.310 | 0.796 | -0.422 | -0.423 | 0.285 | 0.578 |
| *Hypostomus argus* | 0.849 | 1.174 | -1.127 | -0.467 | 0.088 | 0.424 |
| *Leporinus friderici* | 0.129 | 0.191 | 0.139 | -0.065 | 0.898 | -0.264 |
| *Loricariichthys typus* | -0.205 | 1.027 | -0.024 | -0.240 | -0.247 | -0.030 |
| *Markiana geayi* | 0.071 | -0.415 | 0.366 | -0.977 | 0.997 | -0.372 |
| *Microglanis iheringi* | 0.225 | 0.628 | -0.313 | 0.897 | -0.531 | 0.453 |
| *Ochmacanthus alternus* | -0.711 | 1.212 | 0.444 | 0.476 | 1.008 | 0.119 |
| *Odontostilbe pulcher* | -0.164 | -0.204 | 0.756 | -0.585 | -0.637 | 0.408 |
| *Otocinclus sp.* | 0.038 | 0.848 | 0.498 | -0.234 | 0.038 | 0.207 |
| *Parauchenipterus galeatus* | 0.084 | -0.131 | -0.610 | 0.888 | -0.104 | 0.995 |
| *Pimelodella sp.2* | -0.143 | 0.486 | -0.152 | -0.043 | -0.036 | -0.221 |
| *Pimelodella sp.3* | -0.060 | 0.570 | -0.093 | -0.335 | -1.250 | 0.186 |
| *Poecilia reticulata* | 0.067 | -0.100 | -0.661 | 0.965 | -0.410 | 0.838 |
| *Prochilodus mariae* | 0.201 | 0.073 | 1.062 | -0.354 | 0.560 | -0.291 |
| *Pterygoplichthys multirad.* | 0.728 | 1.088 | -0.606 | -0.626 | -0.565 | 0.270 |
| *Pygocentrus cariba* | 0.375 | -0.937 | 0.819 | 0.300 | 0.622 | 1.322 |
| *Pyrrhulina lugubris* | -0.134 | -0.339 | -0.096 | 0.431 | -1.289 | 0.169 |
| *Rachovia maculipinnus* | 0.150 | -0.490 | -0.913 | 0.350 | -0.931 | 0.430 |
| *Rhamdia sp.* | -0.237 | 0.744 | 0.162 | 0.262 | 0.054 | 0.197 |
| *Rineloricaria caracasensis* | -0.330 | 0.854 | -0.529 | -0.819 | -0.340 | -0.491 |
| *Roeboides dayi* | -0.146 | -0.453 | 0.749 | -0.505 | -0.435 | -0.558 |
| *Schizodon isognathus* | -0.063 | 0.050 | 0.578 | 0.190 | -0.093 | 0.131 |
| *Serrasalmus irritans* | 0.093 | -0.972 | 0.744 | 0.297 | 1.017 | 0.792 |
| *Serrasalmus medinai* | 0.684 | -1.051 | -0.126 | 0.766 | 1.314 | 0.665 |
| *Steindachnerina argentea* | 0.263 | 0.115 | 1.117 | -0.534 | -0.366 | -0.187 |
| *Synbranchus marmoratus* | -1.923 | 0.491 | 0.426 | -0.108 | 0.379 | -0.533 |
| *Tetragonopterus argenteus* | 0.345 | -0.683 | 0.859 | -0.929 | -0.017 | 0.023 |
| *Thoracocharax stellatus* | 0.391 | -1.781 | -1.609 | -1.991 | 0.703 | 0.700 |
| *Triportheus sp.* | -0.105 | -1.026 | -0.745 | -0.636 | 0.245 | -0.399 |
| Eigenvalue | 5.368 | 2.118 | 0.723 | 0.533 | 0.338 | 0.299 |

Table S9: The eigenvalues, proportion of variance explained, eigenvectors (variable scores), and species scores from principal component analysis yielding species ordination of the life history data. Species scores are weighted by sums of species scores.

|  | PC1 | PC2 | PC3 | PC4 | PC5 | PC6 |
| --- | --- | --- | --- | --- | --- | --- |
| Eigenvalue | 2.882 | 2.120 | 0.825 | 0.585 | 0.301 | 0.190 |
| Proportion Explained | 0.412 | 0.303 | 0.118 | 0.084 | 0.043 | 0.027 |
| Cumulative Proportion | 0.412 | 0.715 | 0.833 | 0.916 | 0.959 | 0.986 |
| Variable scores | PC1 | PC2 | PC3 | PC4 | PC5 | PC6 |
| Generation time | -1.412 | 0.193 | -0.539 | 0.100 | -0.671 | 0.017 |
| Reproductive season | 1.222 | 0.698 | 0.731 | 0.319 | -0.253 | -0.222 |
| Reproductive bouts | 1.320 | 0.339 | -0.018 | -0.853 | -0.366 | 0.282 |
| Fecundity | -1.267 | -0.032 | 1.003 | -0.066 | 0.052 | 0.423 |
| Egg diameter | -0.232 | 1.466 | -0.531 | -0.253 | 0.423 | 0.111 |
| Parental care | 0.209 | 1.488 | -0.017 | 0.645 | -0.108 | 0.206 |
| Body size | -1.071 | 0.970 | 0.448 | -0.562 | -0.029 | -0.413 |
| Species Scores | PC1 | PC2 | PC3 | PC4 | PC5 | PC6 |
| *Adontosternarchus devananzii* | -0.545 | -0.162 | -0.390 | 0.004 | 0.659 | -1.626 |
| *Aequidens pulcher* | 0.407 | 0.930 | 0.564 | 0.908 | -0.322 | 0.451 |
| *Ancistrus sp.* | 0.359 | 1.097 | -1.204 | -0.028 | 0.201 | 0.531 |
| *Aphyocharax alburnus* | 0.209 | -0.587 | -0.143 | -0.242 | -0.908 | 0.912 |
| *Apistogramma hoignei* | 0.994 | 0.057 | 0.534 | 1.760 | 0.124 | -0.829 |
| *Astronotus ocellatus* | -0.147 | 1.249 | 0.215 | 0.473 | -0.618 | 0.862 |
| *Astyanax bimaculatus* | -0.614 | -0.492 | 0.177 | 0.366 | 0.409 | -0.037 |
| *Brachyhypopomus sp.* | -0.227 | -0.308 | -0.175 | -0.758 | -0.607 | -0.897 |
| *Bryconamericus beta* | 1.062 | -0.614 | 1.517 | -0.182 | -1.175 | -0.098 |
| *Bunocephalus amaurus* | 0.185 | -0.327 | -0.501 | -0.497 | -0.781 | 0.118 |
| *Caquetaia kraussii* | 0.183 | 1.192 | 1.201 | 0.521 | -0.363 | -0.007 |
| *Characidium sp.* | 0.225 | -0.657 | -0.356 | 0.672 | -0.519 | -0.271 |
| *Charax gibbosus* | 0.479 | -0.119 | 0.432 | -0.595 | -0.049 | -0.776 |
| *Cheirodontops geayi* | -0.062 | -0.839 | -0.021 | 0.853 | 1.376 | 0.440 |
| *Cichlasoma orinocense* | -0.075 | 0.877 | 0.188 | 1.026 | -0.305 | 0.454 |
| *Corydoras aeneus* | -0.227 | -0.187 | -0.943 | 0.975 | 0.648 | -0.156 |
| *Corydoras habrosus* | 0.552 | -0.370 | -1.010 | 0.748 | -1.120 | -0.402 |
| *Corydoras septemtrionalis* | 0.035 | -0.109 | -0.846 | 0.000 | -0.274 | 0.459 |
| *Crenicichla saxatilis* | 0.053 | 0.881 | -0.163 | 1.041 | -0.392 | -0.325 |
| *Ctenobrycon spilurus* | 0.039 | -0.502 | -0.058 | -0.126 | -0.569 | 0.409 |
| *Eigenmannia virescens* | -0.244 | -0.076 | -0.421 | -0.883 | -0.104 | -0.624 |
| *Entomocorus gameroi* | -0.083 | -0.604 | -0.638 | 0.232 | -0.142 | 0.060 |
| *Gephyrocharax valenciae* | 0.738 | -0.474 | 0.575 | -0.469 | 0.292 | 0.845 |
| *Gymnotus carapo* | -0.157 | 0.643 | 0.108 | -0.607 | -0.257 | -1.015 |
| *Hemigrammus sp.* | 0.888 | -0.419 | 0.613 | 0.009 | 0.208 | -0.040 |
| *Hoplias malabaricus* | -0.160 | 0.745 | 0.575 | -0.942 | -0.563 | -0.421 |
| *Hoplosternum littorale* | -0.544 | 0.650 | -0.092 | 0.045 | 0.418 | 0.598 |
| *Hypoptopoma sp.* | 0.159 | -0.170 | -1.161 | -0.743 | -0.191 | 0.032 |
| *Hypostomus argus* | -0.091 | 1.089 | -0.552 | -0.201 | 0.300 | 0.005 |
| *Leporinus friderici* | -1.042 | -0.249 | 1.085 | -0.163 | 0.553 | 0.290 |
| *Loricariichthys typus* | 0.025 | 1.140 | -0.399 | -0.791 | -0.224 | 0.508 |
| *Markiana geayi* | -0.606 | -0.441 | 0.093 | 0.331 | 0.488 | -0.126 |
| *Microglanis iheringi* | 0.053 | -0.407 | -0.350 | -0.074 | -0.393 | 0.783 |
| *Ochmacanthus alternus* | 0.032 | -0.598 | -0.425 | 0.423 | -0.417 | -0.140 |
| *Odontostilbe pulcher* | 0.540 | -0.539 | 0.668 | 0.071 | 0.754 | 0.493 |
| *Otocinclus sp.* | 0.634 | -0.498 | -0.582 | -0.064 | -0.363 | 0.127 |
| *Parauchenipterus galeatus* | -0.385 | -0.027 | -0.490 | -0.456 | 0.615 | -0.129 |
| *Pimelodella sp.2* | -0.416 | -0.528 | 0.203 | -0.073 | -0.173 | 0.577 |
| *Pimelodella sp.3* | -0.315 | -0.512 | -0.101 | -0.012 | -0.103 | 0.367 |
| *Poecilia reticulata* | 1.462 | 0.540 | 0.074 | 0.802 | 1.397 | -0.093 |
| *Prochilodus mariae* | -1.344 | -0.196 | 0.868 | -0.098 | -0.594 | 0.519 |
| *Pterygoplichthys multirad.* | -0.280 | 1.120 | -0.482 | -0.431 | 0.562 | 0.439 |
| *Pygocentrus cariba* | -0.548 | 0.524 | 0.184 | -0.400 | 0.528 | 0.015 |
| *Pyrrhulina lugubris* | 0.117 | -0.500 | -0.783 | 0.409 | -0.220 | -0.397 |
| *Rachovia maculipinnus* | 1.765 | -0.199 | -0.206 | -1.620 | 1.434 | 0.373 |
| *Rhamdia sp.* | -0.832 | -0.270 | 0.530 | -0.032 | 0.536 | -0.344 |
| *Rineloricaria caracasensis* | 0.079 | 0.359 | -0.222 | -0.119 | -0.420 | -0.472 |
| *Roeboides dayi* | 1.093 | -0.025 | 1.052 | -0.715 | 0.192 | -0.587 |
| *Schizodon isognathus* | -0.944 | -0.243 | 0.812 | -0.143 | 0.513 | -0.225 |
| *Serrasalmus irritans* | -0.288 | 0.240 | 0.178 | -0.412 | -0.224 | 0.586 |
| *Serrasalmus medinai* | -0.280 | 0.230 | 0.167 | -0.391 | -0.222 | 0.633 |
| *Steindachnerina argentea* | -0.312 | -0.583 | 0.533 | 0.089 | -0.689 | -0.028 |
| *Synbranchus marmoratus* | -0.170 | 0.474 | -0.013 | -0.149 | -0.295 | -2.171 |
| *Tetragonopterus argenteus* | -0.585 | -0.467 | 0.063 | 0.384 | 0.493 | -0.006 |
| *Thoracocharax stellatus* | -0.173 | -0.630 | -0.361 | 0.223 | -0.153 | 0.509 |
| *Triportheus sp.* | -0.673 | -0.111 | -0.124 | 0.052 | 1.047 | -0.153 |

Table S10: The eigenvalues, proportion of variance explained, eigenvectors (variable scores), and species scores from principal component analysis yielding species ordination of the trophic data. Species scores are weighted by sums of species scores.

|  | PC1 | PC2 | PC3 | PC4 | PC5 | PC6 |
| --- | --- | --- | --- | --- | --- | --- |
| Eigenvalue | 2.125 | 1.738 | 1.434 | 1.156 | 0.951 | 0.907 |
| Proportion | 0.213 | 0.174 | 0.143 | 0.116 | 0.095 | 0.091 |
| Cumulative | 0.213 | 0.386 | 0.530 | 0.645 | 0.740 | 0.831 |
| Variable scores | PC1 | PC2 | PC3 | PC4 | PC5 | PC6 |
| Detritus | 0.998 | -0.542 | 0.004 | 0.505 | -0.840 | 0.114 |
| Algae | 1.165 | -0.013 | 0.055 | -0.367 | 0.389 | -0.282 |
| Macrophytes | -0.223 | -0.265 | -1.249 | -0.177 | 0.614 | 0.372 |
| Microorganisms | 0.891 | 0.356 | 0.281 | -0.643 | 0.295 | -0.545 |
| Worms mollusks | -0.422 | -0.211 | -0.826 | 0.285 | -0.174 | -1.063 |
| Micro-crustaceans | -0.060 | 1.131 | 0.220 | 0.377 | 0.371 | -0.033 |
| Decapod crustaceans | -0.723 | -0.637 | 0.477 | -0.355 | -0.037 | -0.595 |
| Aquatic insects | -0.676 | 1.092 | 0.140 | 0.170 | -0.334 | -0.154 |
| Terrestrial insects | -0.475 | 0.149 | -0.099 | -1.210 | -0.598 | 0.277 |
| Fish | -0.632 | -0.801 | 0.856 | 0.183 | 0.521 | 0.111 |
| Species scores | PC1 | PC2 | PC3 | PC4 | PC5 | PC6 |
| *Adontosternarchus devanan.* | -0.299 | 1.074 | 0.249 | 0.622 | -0.075 | 0.109 |
| *Aequidens pulcher* | -0.493 | -0.296 | -1.652 | 0.627 | -0.255 | -1.827 |
| *Ancistrus sp.* | 0.886 | -0.603 | 0.067 | 0.617 | -1.066 | 0.382 |
| *Aphyocharax alburnus* | -0.226 | 1.226 | 0.336 | 0.568 | 0.277 | 0.204 |
| *Apistogramma hoignei* | -0.218 | 0.814 | -0.330 | 0.606 | 0.439 | -0.230 |
| *Astronotus ocellatus* | -1.203 | -1.069 | 0.454 | -1.627 | -0.758 | -1.315 |
| *Astyanax bimaculatus* | 0.077 | -0.090 | -0.827 | -0.583 | 0.524 | 0.694 |
| *Brachyhypopomus sp.* | -0.303 | 1.136 | 0.283 | 0.657 | -0.050 | 0.130 |
| *Bryconamericus beta* | -0.026 | 0.100 | -0.744 | -0.340 | 0.534 | 0.604 |
| *Bunocephalus amaurus* | -0.478 | 0.104 | 0.066 | 0.076 | -0.225 | -0.166 |
| *Caquetaia kraussii* | -0.619 | -0.351 | 0.550 | -0.212 | -0.017 | 0.036 |
| *Characidium sp.* | -0.369 | 0.968 | 0.209 | 0.459 | -0.314 | 0.055 |
| *Charax gibbosus* | -0.597 | -0.751 | 0.978 | 0.231 | 0.517 | 0.054 |
| *Cheirodontops geayi* | 0.359 | 1.203 | 0.393 | 0.370 | 1.038 | -0.114 |
| *Cichlasoma orinocense* | -0.232 | -0.282 | -1.546 | 0.387 | 0.289 | -1.014 |
| *Corydoras aeneus* | 1.032 | 0.791 | 0.506 | -0.869 | 0.758 | -1.109 |
| *Corydoras habrosus* | 1.795 | 0.264 | 0.460 | -1.384 | 1.245 | -1.324 |
| *Corydoras septentrionalis* | -0.049 | 0.641 | 0.236 | 0.304 | -0.557 | -0.046 |
| *Crenicichla saxatilis* | -0.476 | -0.116 | 0.690 | 0.440 | 0.254 | 0.330 |
| *Ctenobrycon spilurus* | -0.039 | -0.091 | -0.813 | 0.084 | 0.787 | 0.718 |
| *Eigenmannia virescens* | -0.173 | 1.312 | 0.354 | 0.733 | 0.372 | 0.114 |
| *Entomocorus gameroi* | 0.435 | 0.609 | 0.364 | -1.596 | -0.150 | -0.498 |
| *Gephyrocharax valenciae* | -0.297 | 0.464 | -0.234 | -1.073 | -0.469 | 0.532 |
| *Gymnotus carapo* | -0.852 | -0.576 | 0.623 | -0.023 | 0.009 | -0.902 |
| *Hemigrammus sp.* | -0.060 | 0.833 | 0.153 | -0.271 | -0.081 | 0.079 |
| *Hoplias malabaricus* | -0.378 | -0.870 | 0.892 | 0.387 | 0.831 | 0.533 |
| *Hoplosternum littorale* | -0.167 | -0.098 | -0.850 | 0.644 | -0.857 | -1.270 |
| *Hypoptopoma sp.* | 1.213 | -0.505 | 0.104 | 0.205 | -0.445 | 0.067 |
| *Hypostomus argus* | 0.815 | -0.571 | 0.064 | 0.588 | -0.997 | 0.384 |
| *Leporinus friderici* | -0.209 | -0.719 | -0.723 | 0.127 | 0.767 | -0.277 |
| *Loricariichthys typus* | 0.627 | -0.155 | 0.057 | 0.415 | -0.680 | 0.136 |
| *Markiana geayi* | -0.068 | -0.356 | -1.208 | -0.264 | 0.748 | 0.864 |
| *Microglanis iheringi* | -0.414 | 0.822 | 0.186 | 0.371 | -0.488 | 0.085 |
| *Ochmacanthus alternus* | -0.273 | -0.898 | 0.886 | 0.421 | 0.725 | 0.531 |
| *Odontostilbe pulcher* | 1.342 | 0.233 | 0.197 | -0.454 | 1.164 | -0.468 |
| *Otocinclus sp.* | 1.073 | -0.510 | 0.123 | 0.333 | -0.738 | 0.136 |
| *Parauchenipterus galeatus* | -0.483 | 0.258 | -0.100 | -1.077 | -0.860 | 0.669 |
| *Pimelodella sp.2* | -0.352 | -0.230 | -1.135 | 0.444 | -0.181 | -1.149 |
| *Pimelodella sp.3* | -0.538 | 0.222 | -0.104 | 0.365 | -0.318 | -1.014 |
| *Poecilia reticulata* | 1.268 | -0.173 | 0.217 | -0.261 | 0.219 | -0.322 |
| *Prochilodus mariae* | 0.874 | -0.557 | 0.064 | 0.549 | -0.937 | 0.345 |
| *Pterygoplichthys multirad.* | 0.920 | -0.558 | 0.066 | 0.504 | -0.836 | 0.320 |
| *Pygocentrus cariba* | -0.853 | -1.409 | 1.126 | -0.263 | 0.553 | -0.619 |
| *Pyrrhulina lugubris* | -0.301 | 0.249 | -0.096 | -1.691 | -1.065 | 0.736 |
| *Rachovia maculipinnus* | -0.426 | 0.859 | 0.024 | 0.017 | -0.470 | 0.059 |
| *Rhamdia sp.* | -0.555 | -0.637 | 0.569 | -0.134 | 0.261 | -0.024 |
| *Rineloricaria caracasensis* | 0.846 | -0.421 | 0.076 | 0.478 | -0.811 | 0.265 |
| *Roeboides dayi* | -0.448 | 0.458 | 0.510 | 0.492 | 0.091 | 0.139 |
| *Schizodon isognathus* | -0.080 | -0.467 | -1.453 | -0.105 | 1.124 | 1.047 |
| *Serrasalmus irritans* | -0.284 | -0.789 | 0.726 | 0.382 | 0.709 | 0.542 |
| *Serrasalmus medinai* | -0.323 | -0.299 | 0.639 | 0.408 | 0.439 | 0.397 |
| *Steindachnerina argentea* | 0.988 | -0.470 | 0.046 | 0.241 | -0.443 | 0.163 |
| *Synbranchus marmoratus* | -0.569 | 0.227 | 0.309 | 0.212 | -0.266 | -0.221 |
| *Tetragonopterus argenteus* | -0.071 | -0.119 | -0.864 | -0.122 | 0.553 | 0.710 |
| *Thoracocharax stellatus* | -0.507 | 0.378 | -0.098 | -1.423 | -1.150 | 0.770 |
| *Triportheus sp.* | -0.243 | -0.206 | -1.071 | -0.594 | 0.336 | 0.971 |

Table S11: The eigenvalues, proportion of variance explained, eigenvectors (variable scores), and species scores from principal component analysis yielding species ordination of the defense data. Species scores are weighted by sums of species scores.

|  | PC1 | PC2 | PC3 | PC4 | PC5 | PC6 |
| --- | --- | --- | --- | --- | --- | --- |
| Eigenvalue | 2.608 | 1.755 | 1.186 | 0.510 | 0.437 | 0.289 |
| Proportion Explained | 0.373 | 0.251 | 0.170 | 0.073 | 0.062 | 0.041 |
| Cumulative Proportion | 0.373 | 0.623 | 0.793 | 0.866 | 0.928 | 0.969 |
| Variable scores | PC1 | PC2 | PC3 | PC4 | PC5 | PC6 |
| Spines | 1.339 | -0.202 | 0.631 | -0.314 | 0.254 | 0.640 |
| Venom | -0.027 | 0.649 | 1.432 | -0.308 | -0.382 | -0.288 |
| Armor | 1.272 | 0.588 | 0.286 | 0.748 | 0.161 | -0.175 |
| Aggression | 0.236 | -1.434 | 0.370 | -0.264 | 0.531 | -0.433 |
| Crypsis | 1.268 | -0.128 | -0.672 | -0.553 | -0.561 | -0.151 |
| Speed | -1.419 | -0.476 | 0.372 | 0.026 | -0.216 | 0.268 |
| Body diameter | 0.471 | -1.346 | 0.258 | 0.549 | -0.588 | 0.070 |
| Species Scores | PC1 | PC2 | PC3 | PC4 | PC5 | PC6 |
| *Adontosternarchus devananzii* | 0.136 | -0.066 | -0.837 | -0.695 | -1.424 | -0.635 |
| *Aequidens pulcher* | 0.464 | -0.904 | 0.081 | -0.989 | 0.233 | 0.194 |
| *Ancistrus sp.* | 1.183 | 0.302 | -0.069 | 0.526 | 0.184 | 0.541 |
| *Aphyocharax alburnus* | -0.609 | 0.259 | -0.257 | -0.046 | 0.318 | 0.082 |
| *Apistogramma hoignei* | 0.292 | 0.162 | -0.284 | -1.187 | 0.035 | 1.230 |
| *Astronotus ocellatus* | 0.555 | -1.291 | 0.191 | -0.447 | -0.445 | 0.316 |
| *Astyanax bimaculatus* | -0.528 | -0.085 | -0.159 | 0.436 | -0.284 | 0.191 |
| *Brachyhypopomus sp.* | -0.086 | 0.128 | -0.671 | -0.522 | -0.648 | -0.491 |
| *Bryconamericus beta* | -0.579 | 0.131 | -0.220 | 0.133 | 0.094 | 0.123 |
| *Bunocephalus amaurus* | 0.944 | 0.325 | -0.601 | -0.641 | -0.337 | -0.073 |
| *Caquetaia kraussii* | 0.523 | -1.154 | 0.152 | -0.638 | -0.206 | 0.273 |
| *Characidium sp.1* | -0.146 | 0.383 | -0.744 | -0.879 | -0.201 | -0.571 |
| *Charax gibbosus* | -0.511 | -0.158 | -0.138 | 0.539 | -0.413 | 0.214 |
| *Cheirodontops geayi* | -0.627 | 0.336 | -0.279 | -0.155 | 0.454 | 0.058 |
| *Cichlasoma orinocense* | 0.498 | -1.047 | 0.122 | -0.789 | -0.017 | 0.239 |
| *Corydoras aeneus* | 0.151 | 0.836 | 1.456 | 0.292 | -0.256 | -0.877 |
| *Corydoras habrosus* | 0.084 | 1.119 | 1.376 | -0.106 | 0.240 | -0.966 |
| *Corydoras septemtrionalis* | 0.153 | 0.826 | 1.459 | 0.305 | -0.274 | -0.874 |
| *Crenicichla saxatilis* | -0.049 | -0.813 | 0.507 | -0.459 | 1.130 | 0.780 |
| *Ctenobrycon spilurus* | -0.557 | 0.036 | -0.194 | 0.266 | -0.071 | 0.152 |
| *Eigenmannia virescens* | -0.550 | 0.006 | -0.185 | 0.309 | -0.125 | 0.162 |
| *Entomocorus gameroi* | -0.113 | 0.261 | -0.017 | -0.435 | 0.821 | 1.190 |
| *Gephyrocharax valenciae* | -0.621 | 0.307 | -0.270 | -0.114 | 0.404 | 0.067 |
| *Gymnotus carapo* | -0.063 | 0.028 | -0.643 | -0.381 | -0.824 | -0.459 |
| *Hemigrammus sp.* | -0.611 | 0.266 | -0.259 | -0.056 | 0.331 | 0.080 |
| *Hoplias malabaricus* | 0.356 | -0.707 | -0.498 | -0.472 | -0.034 | -2.051 |
| *Hoplosternum littorale* | 0.839 | 0.217 | 0.269 | 1.507 | 0.677 | 0.639 |
| *Hypoptopoma sp.* | 1.120 | 0.570 | -0.145 | 0.149 | 0.655 | 0.456 |
| *Hypostomus argus* | 1.235 | 0.081 | -0.006 | 0.835 | -0.203 | 0.610 |
| *Leporinus friderici* | -0.506 | -0.177 | -0.133 | 0.566 | -0.446 | 0.220 |
| *Loricariichthys typus* | 1.135 | 0.169 | -0.514 | 0.674 | -0.895 | -0.767 |
| *Markiana geayi* | -0.508 | -0.169 | -0.135 | 0.554 | -0.432 | 0.217 |
| *Microglanis iheringi* | 0.468 | 0.455 | -0.577 | -1.294 | 0.430 | 0.327 |
| *Ochmacanthus alternus* | -0.189 | 0.663 | -0.572 | -0.333 | 1.043 | -0.760 |
| *Odontostilbe pulcher* | -0.612 | 0.269 | -0.260 | -0.061 | 0.337 | 0.079 |
| *Otocinclus sp.* | 0.378 | 0.879 | -0.183 | 0.506 | 1.570 | -0.337 |
| *Parauchenipterus galeatus* | 0.966 | 0.086 | -0.166 | -0.087 | -0.092 | 0.845 |
| *Pimelodella sp.2* | -0.267 | 0.478 | 1.495 | -0.941 | -0.544 | 0.381 |
| *Pimelodella sp.3* | -0.282 | 0.544 | 1.477 | -1.034 | -0.428 | 0.360 |
| *Poecilia reticulata* | -0.485 | 0.540 | -0.411 | -0.338 | 0.818 | -0.270 |
| *Prochilodus mariae* | -0.474 | -0.314 | -0.094 | 0.758 | -0.686 | 0.263 |
| *Pterygoplichthys multirad.* | 1.231 | 0.099 | -0.011 | 0.810 | -0.172 | 0.604 |
| *Pygocentrus cariba* | 0.098 | -1.320 | 0.648 | 0.716 | 1.036 | -0.966 |
| *Pyrrhulina lugubris* | -0.616 | 0.288 | -0.265 | -0.087 | 0.370 | 0.073 |
| *Rachovia maculipinnus* | -0.625 | 0.324 | -0.275 | -0.137 | 0.432 | 0.062 |
| *Rhamdia sp.* | -0.003 | 0.170 | 1.509 | -0.404 | -1.079 | 0.214 |
| *Rineloricaria caracasensis* | 1.064 | 0.469 | -0.599 | 0.252 | -0.369 | -0.862 |
| *Roeboides dayi* | -0.548 | 0.000 | -0.183 | 0.317 | -0.135 | 0.164 |
| *Schizodon isognathus* | -0.478 | -0.299 | -0.098 | 0.737 | -0.660 | 0.258 |
| *Serrasalmus irritans* | -0.107 | -1.585 | 0.564 | 0.174 | 0.674 | -0.645 |
| *Serrasalmus medinai* | -0.134 | -1.471 | 0.532 | 0.013 | 0.874 | -0.681 |
| *Steindachnerina argentea* | -0.528 | -0.086 | -0.159 | 0.438 | -0.286 | 0.191 |
| *Synbranchus marmoratus* | -0.081 | 0.205 | -0.442 | 0.309 | 0.241 | -0.616 |
| *Tetragonopterus argenteus* | -0.518 | -0.126 | -0.147 | 0.495 | -0.357 | 0.204 |
| *Thoracocharax stellatus* | -0.672 | -0.049 | -0.117 | 0.312 | -0.225 | 0.367 |
| *Triportheus sp.* | -0.590 | -0.394 | -0.019 | 0.797 | -0.831 | 0.475 |

Table S12: The eigenvalues, proportion of variance explained, eigenvectors (variable scores), and species scores from principal component analysis yielding species ordination of the metabolic data. Species scores are weighted by sums of species scores.

|  | PC1 | PC2 | PC3 | PC4 |
| --- | --- | --- | --- | --- |
| Eigenvalue | 1.507 | 1.358 | 0.745 | 0.390 |
| Proportion Explained | 0.377 | 0.339 | 0.186 | 0.098 |
| Cumulative Proportion | 0.377 | 0.716 | 0.902 | 1.000 |
| Variable scores | PC1 | PC2 | PC3 | PC4 |
| Activity level | -0.950 | -1.188 | 1.110 | -0.403 |
| Hypoxia tolerance | 1.270 | -1.188 | 0.357 | 0.747 |
| Visceral fat storage | -1.739 | -0.030 | -0.198 | 0.802 |
| Aerial respiration | 0.220 | 1.487 | 1.168 | 0.291 |
| Species Scores | PC1 | PC2 | PC3 | PC4 |
| *Adontosternarchus devananzii* | 0.632 | -0.781 | -0.016 | 0.439 |
| *Aequidens pulcher* | -0.337 | -0.166 | -0.442 | -0.263 |
| *Ancistrus sp.* | 0.545 | 0.796 | 0.210 | -0.544 |
| *Aphyocharax alburnus* | -0.585 | 0.051 | 0.493 | 0.058 |
| *Apistogramma hoignei* | 0.033 | -0.159 | -0.357 | -0.921 |
| *Astronotus ocellatus* | -0.163 | -0.162 | -0.402 | -0.571 |
| *Astyanax bimaculatus* | -0.585 | 0.051 | 0.493 | 0.058 |
| *Brachyhypopomus sp.* | -0.246 | 0.514 | 0.532 | 0.200 |
| *Bryconamericus beta* | 0.148 | -0.363 | 0.799 | 0.393 |
| *Bunocephalus amaurus* | 0.131 | 0.484 | -1.548 | 0.504 |
| *Caquetaia kraussii* | -0.492 | -0.169 | -0.477 | 0.013 |
| *Characidium sp.1* | -0.297 | 0.101 | -0.936 | 0.331 |
| *Charax gibbosus* | 0.069 | -0.587 | -0.211 | 0.657 |
| *Cheirodontops geayi* | -0.585 | 0.051 | 0.493 | 0.058 |
| *Cichlasoma orinocense* | -0.401 | 0.511 | 0.496 | 0.475 |
| *Corydoras aeneus* | -0.207 | 0.781 | 0.037 | 0.794 |
| *Corydoras habrosus* | 0.122 | 0.787 | 0.113 | 0.210 |
| *Corydoras septemtrionalis* | -0.207 | 0.781 | 0.037 | 0.794 |
| *Crenicichla saxatilis* | 0.859 | -0.777 | 0.036 | 0.035 |
| *Ctenobrycon spilurus* | -0.430 | 0.054 | 0.529 | -0.218 |
| *Eigenmannia virescens* | -0.337 | -0.166 | -0.442 | -0.263 |
| *Entomocorus gameroi* | 0.069 | -0.587 | -0.211 | 0.657 |
| *Gephyrocharax valenciae* | -0.585 | 0.051 | 0.493 | 0.058 |
| *Gymnotus carapo* | -0.073 | 0.518 | 0.572 | -0.109 |
| *Hemigrammus sp.* | -0.025 | -0.367 | 0.759 | 0.702 |
| *Hoplias malabaricus* | 0.034 | 0.910 | -0.969 | 1.072 |
| *Hoplosternum littorale* | -0.401 | 0.511 | 0.496 | 0.475 |
| *Hypoptopoma sp.* | 1.219 | 0.380 | 0.502 | -0.101 |
| *Hypostomus argus* | 0.545 | 0.796 | 0.210 | -0.544 |
| *Leporinus friderici* | 0.263 | -0.788 | -0.101 | 1.097 |
| *Loricariichthys typus* | 0.545 | 0.796 | 0.210 | -0.544 |
| *Markiana geayi* | -0.585 | 0.051 | 0.493 | 0.058 |
| *Microglanis iheringi* | -0.297 | 0.101 | -0.936 | 0.331 |
| *Ochmacanthus alternus* | 0.45429 | 0.11558 | -0.76356 | -1.00632 |
| *Odontostilbe pulcher* | -0.43012 | 0.05405 | 0.52871 | -0.2181 |
| *Otocinclus sp.* | -0.14237 | 0.10398 | -0.90074 | 0.055293 |
| *Parauchenipterus galeatus* | -0.4917 | -0.16877 | -0.47723 | 0.012776 |
| *Pimelodella sp.2* | -0.6424 | -0.378 | -0.12108 | -0.23418 |
| *Pimelodella sp.3* | -0.6424 | -0.378 | -0.12108 | -0.23418 |
| *Poecilia reticulata* | 0.1093 | -0.36338 | 0.05174 | -1.57164 |
| *Prochilodus mariae* | -0.48736 | -0.37498 | -0.08544 | -0.51004 |
| *Pterygoplichthys multirad.* | 0.31818 | 0.79118 | 0.15769 | -0.14 |
| *Pygocentrus cariba* | 0.43839 | -0.57946 | -0.1263 | -0.00142 |
| *Pyrrhulina lugubris* | -0.48736 | -0.37498 | -0.08544 | -0.51004 |
| *Rachovia maculipinnus* | 0.63242 | -0.781 | -0.01605 | 0.438944 |
| *Rhamdia sp.* | -0.14237 | 0.10398 | -0.90074 | 0.055293 |
| *Rineloricaria caracasensis* | 0.31818 | 0.79118 | 0.15769 | -0.14 |
| *Roeboides dayi* | -0.16333 | -0.16239 | -0.40174 | -0.57147 |
| *Schizodon isognathus* | 0.28769 | -0.78869 | 0.22984 | -0.24837 |
| *Serrasalmus irritans* | 0.85926 | -0.77659 | 0.0361 | 0.03534 |
| *Serrasalmus medinai* | 0.63242 | -0.781 | -0.01605 | 0.438944 |
| *Steindachnerina argentea* | -0.31404 | -0.37162 | -0.04559 | -0.81842 |
| *Synbranchus marmoratus* | 1.82074 | 0.76647 | -0.06986 | -0.23681 |
| *Tetragonopterus argenteus* | 0.53896 | -0.56119 | 0.95425 | 0.483931 |
| *Thoracocharax stellatus* | -0.58516 | 0.05103 | 0.49307 | 0.057762 |
| *Triportheus sp.* | -0.25679 | 0.05742 | 0.56856 | -0.52648 |
