# Supplementary material for: Functional traits, convergent evolution, and periodic tables of niches
Source: Ecol Lett. 2015 Jun 21;18(8):737–51. doi: 10.1111/ele.12462 (PMC4744997; doi:10.1111/ele.12462)
Supplement: Supplementary file 11 [file ELE-18-737-s011.docx]

Table S13: The eigenvalues, proportion of variance explained, eigenvectors (variable scores), and species scores from principal component analysis yielding species ordination within a continuous niche scheme (PCA of the PCAs). Species scores are weighted by sums of species scores.

|  | PC1 | PC2 | PC3 | PC4 | PC5 | PC6 |
| --- | --- | --- | --- | --- | --- | --- |
| Eigenvalue | 1.046 | 0.948 | 0.475 | 0.391 | 0.306 | 0.171 |
| Proportion Explained | 0.285 | 0.258 | 0.129 | 0.106 | 0.083 | 0.047 |
| Cumulative Proportion | 0.285 | 0.543 | 0.672 | 0.779 | 0.862 | 0.909 |
| Variable scores | PC1 | PC2 | PC3 | PC4 | PC5 | PC6 |
| PC1.habitat | 0.365 | 0.507 | -1.044 | 0.070 | -0.222 | 0.027 |
| PC2.habitat | 1.082 | -0.380 | 0.276 | 0.204 | -0.032 | 0.240 |
| PC1.metabolic | 0.295 | 0.249 | 0.377 | -0.312 | 0.682 | -0.158 |
| PC2.metabolic | 0.733 | -0.159 | 0.214 | -0.057 | -0.376 | -0.038 |
| PC1.trophic | 0.803 | -0.470 | -0.499 | 0.263 | 0.466 | -0.412 |
| PC2.trophic | -0.332 | -0.952 | 0.022 | -0.418 | -0.410 | -0.470 |
| PC1.lifehistory | 0.072 | -0.390 | -0.421 | -0.937 | 0.207 | 0.309 |
| PC2.lifehistory | 0.526 | 0.840 | 0.161 | -0.440 | -0.119 | -0.141 |
| PC1.defense | 0.933 | 0.411 | 0.175 | -0.210 | -0.293 | -0.090 |
| PC2.defense | 0.461 | -1.013 | 0.010 | 0.072 | 0.000 | 0.251 |
| Species Scores | PC1 | PC2 | PC3 | PC4 | PC5 | PC6 |
| *Adontosternarchus devananzii* | -0.501 | -0.267 | 1.025 | -0.215 | 0.297 | -1.166 |
| *Aequidens pulcher* | 0.013 | 0.763 | -0.532 | -0.553 | -0.545 | 0.125 |
| *Ancistrus sp.* | 1.180 | 0.309 | -0.177 | -0.354 | 0.121 | -0.043 |
| *Aphyocharax alburnus* | -0.395 | -0.651 | -0.086 | -0.138 | -0.581 | -0.217 |
| *Apistogramma hoignei* | -0.001 | -0.209 | -0.405 | -0.917 | -0.286 | 0.028 |
| *Astronotus ocellatus* | -0.132 | 1.312 | -0.069 | -0.324 | -0.562 | 0.485 |
| *Astyanax bimaculatus* | -0.343 | -0.047 | -0.218 | 0.686 | -0.223 | -0.169 |
| *Brachyhypopomus sp.* | -0.284 | -0.627 | 1.111 | -0.167 | -0.478 | -0.639 |
| *Bryconamericus beta* | -0.307 | -0.322 | -0.410 | -0.472 | 0.546 | 0.601 |
| *Bunocephalus amaurus* | 0.209 | -0.137 | 0.525 | -0.294 | -0.387 | 0.353 |
| *Caquetaia kraussii* | -0.101 | 0.950 | -0.402 | -0.534 | -0.704 | -0.101 |
| *Characidium sp.* | -0.179 | -0.545 | -0.028 | -0.159 | -0.546 | 0.085 |
| *Charax gibbosus* | -0.476 | 0.251 | -0.075 | -0.176 | 0.517 | 0.955 |
| *Cheirodontops geayi* | -0.379 | -0.760 | -0.192 | 0.183 | -0.338 | -0.693 |
| *Cichlasoma orinocense* | 0.200 | 0.752 | -0.384 | -0.200 | -0.784 | -0.282 |
| *Corydoras aeneus* | 0.663 | -0.615 | -0.421 | 0.338 | -0.443 | -0.623 |
| *Corydoras habrosus* | 0.855 | -0.770 | -0.715 | 0.123 | 0.421 | -0.323 |
| *Corydoras septemtrionalis* | 0.445 | -0.411 | -0.276 | -0.010 | -0.799 | 0.207 |
| *Crenicichla saxatilis* | -0.267 | 0.633 | 0.239 | -0.578 | 0.549 | -0.399 |
| *Ctenobrycon spilurus* | -0.315 | -0.165 | -0.198 | 0.254 | -0.032 | 0.266 |
| *Eigenmannia virescens* | -0.464 | -0.618 | 0.892 | -0.132 | -0.264 | -0.796 |
| *Entomocorus gameroi* | -0.170 | -0.345 | -0.372 | 0.157 | 0.220 | -0.539 |
| *Gephyrocharax valenciae* | -0.537 | -0.436 | -0.195 | -0.406 | -0.206 | 0.330 |
| *Gymnotus carapo* | -0.125 | 0.161 | 1.271 | -0.156 | -0.113 | 0.650 |
| *Hemigrammus sp.* | -0.367 | -0.519 | -0.289 | -0.588 | 0.165 | 0.075 |
| *Hoplias malabaricus* | 0.174 | 0.667 | 0.385 | -0.111 | -0.272 | 0.139 |
| *Hoplosternum littorale* | 0.533 | 0.240 | 0.112 | 0.206 | -0.895 | 0.054 |
| *Hypoptopoma sp.* | 0.985 | -0.085 | -0.039 | 0.028 | 0.921 | -0.234 |
| *Hypostomus argus* | 1.217 | 0.417 | -0.095 | -0.074 | -0.041 | -0.228 |
| *Leporinus friderici* | -0.231 | 0.309 | 0.133 | 0.950 | 0.507 | 0.266 |
| *Loricariichthys typus* | 1.007 | 0.167 | 0.506 | -0.359 | -0.018 | -0.413 |
| *Markiana geayi* | -0.327 | 0.065 | -0.152 | 0.719 | -0.195 | 0.100 |
| *Microglanis iheringi* | 0.143 | -0.391 | 0.039 | -0.104 | -0.775 | 0.198 |
| *Ochmacanthus alternus* | 0.288 | -0.275 | 0.682 | 0.445 | 0.641 | 1.522 |
| *Odontostilbe pulcher* | -0.015 | -0.589 | -0.609 | 0.140 | 0.518 | -0.430 |
| *Otocinclus sp.* | 0.627 | -0.483 | -0.357 | 0.210 | 0.521 | 0.715 |
| *Parauchenipterus galeatus* | 0.011 | 0.113 | 0.125 | 0.056 | -0.824 | -0.071 |
| *Pimelodella sp.2* | -0.132 | -0.231 | 0.097 | 0.680 | -0.249 | 0.864 |
| *Pimelodella sp.3* | -0.169 | -0.349 | 0.096 | 0.475 | -0.477 | 0.764 |
| *Poecilia reticulata* | 0.233 | -0.310 | -0.750 | -0.721 | 1.049 | 0.132 |
| *Prochilodus mariae* | -0.041 | 0.167 | -0.248 | 1.381 | 0.238 | -0.616 |
| *Pterygoplichthys multirad.* | 1.187 | 0.399 | -0.069 | 0.081 | -0.144 | -0.372 |
| *Pygocentrus cariba* | -0.413 | 1.232 | 0.057 | 0.174 | 0.371 | 0.253 |
| *Pyrrhulina lugubris* | -0.459 | -0.302 | -0.162 | 0.114 | -0.109 | 0.374 |
| *Rachovia maculipinnus* | -0.469 | -0.412 | -0.474 | -1.406 | 0.614 | 0.451 |
| *Rhamdia sp.* | 0.097 | 0.092 | 0.540 | 0.797 | -0.206 | 0.820 |
| *Rineloricaria caracasensis* | 0.914 | -0.083 | 0.356 | -0.011 | 0.164 | -0.060 |
| *Roeboides dayi* | -0.412 | -0.222 | -0.215 | -0.827 | -0.009 | 0.422 |
| *Schizodon isognathus* | -0.284 | 0.245 | 0.174 | 0.798 | 0.534 | -0.064 |
| *Serrasalmus irritans* | -0.500 | 0.958 | 0.023 | -0.058 | 0.864 | -0.546 |
| *Serrasalmus medinai* | -0.528 | 0.873 | -0.361 | -0.130 | 0.450 | -0.766 |
| *Steindachnerina argentea* | -0.014 | -0.121 | -0.545 | 0.841 | 0.570 | -0.115 |
| *Synbranchus marmoratus* | 0.161 | -0.152 | 1.860 | -0.601 | 0.792 | -0.278 |
| *Tetragonopterus argenteus* | -0.423 | 0.152 | -0.193 | 0.419 | 0.528 | -0.353 |
| *Thoracocharax stellatus* | -0.851 | -0.024 | -0.541 | 0.086 | -0.510 | -0.320 |
| *Triportheus sp.* | -0.531 | 0.245 | 0.009 | 0.435 | -0.104 | -0.381 |
