# Supplementary material for: Functional traits, convergent evolution, and periodic tables of niches
Source: Ecol Lett. 2015 Jun 21;18(8):737–51. doi: 10.1111/ele.12462 (PMC4744997; doi:10.1111/ele.12462)
Supplement: Supplementary file 12 [file ELE-18-737-s012.docx]

Table S14: The eigenvalues, proportion of variance explained, eigenvectors (variable scores), and species scores from principal component analysis yielding species ordination within a continuous niche scheme based on the full set of independent variables as input data. Species scores are weighted by sums of species scores.

|  | PC1 | PC2 | PC3 | PC4 | PC5 | PC6 |
| --- | --- | --- | --- | --- | --- | --- |
| Eigenvalue | 7.535 | 5.815 | 4.763 | 3.417 | 2.276 | 1.998 |
| Proportion Explained | 0.198 | 0.153 | 0.125 | 0.090 | 0.060 | 0.053 |
| Cumulative Proportion | 0.198 | 0.351 | 0.477 | 0.567 | 0.627 | 0.679 |
| Variable scores | PC1 | PC2 | PC3 | PC4 | PC5 | PC6 |
| Relative body depth | 0.238 | 0.926 | 0.208 | -0.022 | 0.117 | -0.029 |
| Relative body width | 0.968 | -0.073 | 0.188 | -0.061 | -0.112 | 0.104 |
| Relative body depth below midline | -0.559 | 0.400 | -0.195 | 0.237 | -0.142 | -0.483 |
| Relative head length | 0.541 | 0.771 | 0.263 | 0.177 | 0.080 | 0.175 |
| Mouth position | 0.439 | -0.601 | 0.187 | -0.514 | 0.069 | -0.002 |
| Relative pectoral length | 0.689 | 0.453 | 0.455 | 0.048 | -0.123 | -0.249 |
| Relative pectoral fin height | 0.846 | 0.336 | 0.062 | 0.209 | -0.217 | 0.032 |
| Relative caudal fin length | 0.726 | 0.237 | 0.633 | 0.037 | 0.028 | 0.002 |
| Relative caudal fin height | 0.680 | 0.638 | 0.274 | -0.172 | 0.310 | -0.036 |
| Relative pelvic fin length | 0.839 | 0.344 | 0.269 | 0.068 | -0.118 | -0.046 |
| Activity level | -0.369 | 0.683 | 0.361 | -0.138 | 0.023 | -0.313 |
| Hypoxia tolerance | -0.206 | 0.292 | -0.254 | 0.175 | 0.518 | 0.141 |
| Visceral fat storage | -0.141 | 0.356 | 0.390 | -0.258 | -0.654 | 0.109 |
| Aerial respiration | 0.245 | -0.457 | 0.082 | -0.231 | 0.086 | -0.371 |
| Detritus | 0.620 | -0.484 | -0.010 | -0.189 | 0.326 | -0.389 |
| Algae | 0.155 | -0.200 | 0.561 | -0.038 | 0.480 | -0.019 |
| Macrophytes | -0.095 | 0.502 | 0.036 | -0.417 | -0.021 | -0.445 |
| Microorganisms | 0.210 | -0.244 | 0.546 | -0.157 | 0.153 | 0.410 |
| Worms mollusks | 0.296 | 0.234 | -0.124 | -0.006 | -0.537 | 0.009 |
| Micro-crustaceans | -0.546 | -0.167 | 0.239 | 0.165 | -0.192 | -0.027 |
| Decapod crustaceans | 0.069 | 0.225 | -0.489 | 0.152 | -0.149 | 0.307 |
| Aquatic insects | -0.400 | -0.207 | 0.064 | 0.338 | -0.619 | 0.161 |
| Terrestrial insects | -0.129 | 0.354 | 0.137 | 0.151 | -0.341 | -0.208 |
| Fish | -0.039 | 0.251 | -0.618 | 0.233 | 0.369 | 0.557 |
| Generation time | 0.228 | -0.063 | -0.494 | -0.744 | -0.188 | 0.122 |
| Reproductive season | 0.166 | 0.071 | 0.043 | 0.855 | 0.110 | -0.094 |
| Reproductive bouts | 0.093 | -0.269 | 0.150 | 0.805 | 0.121 | -0.104 |
| Fecundity | -0.048 | 0.616 | -0.410 | -0.643 | -0.014 | -0.054 |
| Egg diameter | 0.675 | -0.187 | -0.580 | 0.147 | 0.031 | -0.180 |
| Parental care | 0.750 | 0.064 | -0.336 | 0.389 | -0.111 | -0.043 |
| Spines | 0.121 | 0.079 | -0.955 | -0.299 | 0.044 | -0.077 |
| Venom | 0.858 | -0.107 | -0.160 | 0.107 | -0.345 | 0.143 |
| Armor | 0.158 | -0.133 | 0.329 | -0.385 | -0.242 | 0.691 |
| Aggression | 0.733 | -0.603 | 0.053 | -0.164 | 0.118 | -0.107 |
| Crypsis | 0.325 | 0.583 | -0.523 | 0.336 | 0.133 | 0.301 |
| Speed | 0.369 | -0.438 | -0.428 | 0.260 | -0.401 | -0.319 |
| Body diameter | -0.531 | 0.776 | 0.283 | -0.090 | 0.099 | 0.020 |
| Species Scores | PC1 | PC2 | PC3 | PC4 | PC5 | PC6 |
| *Adontosternarchus devananzii* | -1.611 | -0.802 | -1.266 | 0.027 | -0.467 | -0.343 |
| *Aequidens pulcher* | 1.348 | 1.150 | -0.154 | 1.023 | -1.623 | -0.505 |
| *Ancistrus sp.* | 1.921 | -1.185 | -0.119 | 0.330 | 0.516 | -1.108 |
| *Aphyocharax alburnus* | -0.897 | -0.044 | 0.811 | 0.080 | -0.466 | -0.106 |
| *Apistogramma hoignei* | 0.280 | 0.129 | 0.789 | 1.481 | -0.838 | -0.153 |
| *Astronotus ocellatus* | 1.230 | 1.417 | -1.349 | 0.979 | -1.166 | 0.380 |
| *Astyanax bimaculatus* | -0.428 | 0.840 | 0.417 | -1.235 | 0.064 | -0.875 |
| *Brachyhypopomus sp.* | -1.800 | -1.339 | -0.851 | -0.181 | -0.937 | -0.547 |
| *Bryconamericus beta* | -0.631 | 0.518 | 1.009 | 0.771 | 0.866 | -0.777 |
| *Bunocephalus amaurus* | -0.056 | -1.145 | -0.581 | 0.526 | -1.512 | -0.200 |
| *Caquetaia kraussii* | 1.043 | 1.285 | -0.812 | 1.307 | -0.736 | 0.345 |
| *Characidium sp.* | -0.457 | -0.386 | 0.654 | 0.340 | -1.172 | 0.283 |
| *Charax gibbosus* | -0.598 | 0.591 | -0.311 | 0.825 | 0.919 | 0.742 |
| *Cheirodontops geayi* | -1.024 | 0.028 | 1.076 | -0.371 | -0.029 | 0.105 |
| *Cichlasoma orinocense* | 1.390 | 1.012 | -0.291 | 0.129 | -1.295 | -0.762 |
| *Corydoras aeneus* | 0.933 | -0.623 | 1.462 | -1.260 | -0.105 | 2.057 |
| *Corydoras habrosus* | 0.894 | -0.964 | 2.051 | -0.439 | 0.835 | 1.863 |
| *Corydoras septemtrionalis* | 0.922 | -0.458 | 1.012 | -0.567 | -0.784 | 1.510 |
| *Crenicichla saxatilis* | 0.128 | 0.534 | -1.016 | 1.188 | 0.593 | 0.981 |
| *Ctenobrycon spilurus* | -0.562 | 0.482 | 0.524 | -0.566 | 0.228 | -0.825 |
| *Eigenmannia virescens* | -1.826 | -0.937 | -0.691 | -0.196 | -0.585 | 0.028 |
| *Entomocorus gameroi* | -0.036 | 0.194 | 0.871 | -0.074 | -0.192 | 1.030 |
| *Gephyrocharax valenciae* | -1.014 | 0.342 | 0.873 | 0.770 | -0.390 | -0.904 |
| *Gymnotus carapo* | -1.221 | -0.945 | -1.792 | 0.322 | -0.489 | 0.081 |
| *Hemigrammus sp.* | -0.762 | 0.155 | 1.050 | 0.909 | 0.341 | -0.252 |
| *Hoplias malabaricus* | 0.215 | -0.060 | -1.504 | 0.608 | 0.376 | 0.766 |
| *Hoplosternum littorale* | 1.200 | -0.233 | -0.300 | -0.893 | -1.039 | -0.493 |
| *Hypoptopoma sp.* | 1.107 | -1.390 | 0.280 | -0.066 | 1.360 | -0.578 |
| *Hypostomus argus* | 2.048 | -1.011 | -0.377 | -0.190 | 0.417 | -1.124 |
| *Leporinus friderici* | -0.162 | 0.926 | -0.577 | -1.459 | 0.536 | 0.244 |
| *Loricariichthys typus* | 0.959 | -1.574 | -0.888 | 0.018 | 0.356 | -1.083 |
| *Markiana geayi* | -0.366 | 0.874 | 0.184 | -1.427 | 0.009 | -1.109 |
| *Microglanis iheringi* | 0.171 | -0.616 | 0.377 | 0.352 | -1.506 | 0.719 |
| *Ochmacanthus alternus* | -0.596 | -1.149 | -0.375 | -0.197 | 1.003 | 1.489 |
| *Odontostilbe pulcher* | -0.613 | -0.009 | 1.403 | 0.165 | 1.348 | -0.385 |
| *Otocinclus sp.* | 0.434 | -1.167 | 0.952 | 0.040 | 0.759 | 0.199 |
| *Parauchenipterus galeatus* | 0.309 | -0.032 | -0.319 | -0.075 | -1.409 | -0.268 |
| *Pimelodella sp.2* | -0.175 | 0.210 | 0.330 | -1.165 | -1.409 | 0.980 |
| *Pimelodella sp.3* | -0.244 | 0.012 | 0.514 | -0.760 | -1.475 | 1.462 |
| *Poecilia reticulata* | 0.104 | -0.347 | 1.172 | 1.804 | 1.447 | -0.303 |
| *Prochilodus mariae* | 0.090 | 0.598 | -0.301 | -1.989 | 0.696 | -0.427 |
| *Pterygoplichthys multirad.* | 1.908 | -0.971 | -0.391 | -0.421 | 0.393 | -1.114 |
| *Pygocentrus cariba* | 0.455 | 1.455 | -1.525 | 0.183 | 1.247 | 1.290 |
| *Pyrrhulina lugubris* | -0.668 | 0.362 | 0.760 | 0.218 | -0.560 | -0.289 |
| *Rachovia maculipinnus* | -0.553 | 0.101 | 1.089 | 2.506 | 0.300 | -0.230 |
| *Rhamdia sp.* | -0.070 | -0.069 | -0.670 | -1.298 | -0.517 | 1.986 |
| *Rineloricaria caracasensis* | 0.629 | -1.631 | -0.414 | -0.151 | 0.317 | -1.212 |
| *Roeboides dayi* | -0.685 | 0.218 | 0.381 | 1.438 | 0.293 | 0.019 |
| *Schizodon isognathus* | -0.428 | 0.900 | -0.489 | -1.576 | 0.855 | -0.729 |
| *Serrasalmus irritans* | 0.023 | 1.280 | -1.298 | 0.369 | 1.803 | 0.955 |
| *Serrasalmus medinai* | 0.423 | 1.583 | -0.827 | 0.574 | 1.131 | 0.961 |
| *Steindachnerina argentea* | -0.022 | 0.370 | 0.589 | -0.933 | 1.065 | -0.281 |
| *Synbranchus marmoratus* | -1.390 | -1.868 | -1.940 | 0.251 | 0.762 | 0.279 |
| *Tetragonopterus argenteus* | -0.274 | 1.066 | 0.213 | -0.998 | 0.923 | -0.802 |
| *Thoracocharax stellatus* | -0.481 | 1.319 | 0.819 | -0.041 | -0.943 | -1.381 |
| *Triportheus sp.* | -0.514 | 1.003 | -0.233 | -1.002 | -0.115 | -1.589 |
