# Supplementary material for: Functional traits, convergent evolution, and periodic tables of niches
Source: Ecol Lett. 2015 Jun 21;18(8):737–51. doi: 10.1111/ele.12462 (PMC4744997; doi:10.1111/ele.12462)
Supplement: Supplementary file 13 [file ELE-18-737-s013.docx]

**Supplemental Information 1: References for recent syntheses of research on functional traits in ecology or analysis of functional traits to examine community–environment relationships.**

1.

Ackerly, D. (2004). Functional strategies of chaparral shrubs in relation to seasonal water deficit and disturbance. *Ecol. Monogr.* 74, 25–44

2.

Beckman, N.G. & Muller-Landau, H.C. (2011). Linking fruit traits to variation in predispersal vertebrate seed predation, insect seed predation, and pathogen attack. *Ecology* 92, 2131–2140

3.

Bernhardt‐Römermann, M., Gray, A., Vanbergen, A.J., Bergès, L., Bohner, A., Brooker, R.W., *et al.* (2011). Functional traits and local environment predict vegetation responses to disturbance: a pan-European multi-site experiment. *J. Ecol.* 99, 777–787

4.

Cadotte, M., Albert, C.H., & Walker, S.C. (2013). The ecology of differences: assessing community assembly with trait and evolutionary distances. *Ecol. Lett.* 16, 1234-1244

5.

Cornwell, W.K. & Ackerly, D.D. (2009). Community assembly and shifts in plant trait distributions across an environmental gradient in coastal California. *Ecol. Monogr.* 79, 109–126

6.

Franzén, M., Schweiger, O., & Betzholtz, P.E. (2012). Species-area relationships are controlled by species traits. *Plos One* 7(5), 1-10, e 37359

7.

Heino, J., Schmera, D., & Erős, T. (2013). A macroecological perspective of trait patterns in stream communities. *Freshwater* *Biol*. 58:1539–1555

8.

Haddad, N.M., Holyoak, M., Mata, T.M., Davies, K.F., Melbourne, B.A., & Preston, K. (2008) Species' traits predict the effects of disturbance and productivity on diversity. *Ecol. Lett.* 11, 348–356

9.

Laughlin, D.C. (2014a). The intrinsic dimensionality of plant traits and its relevance to community assembly. *J. Ecol.* 102, 186–193

10.

Laughlin, D.C. (2014b). Applying trait-based models to achieve functional targets for theory-driven ecological restoration. *Ecol. Lett.* 17, 771-784

11.

Laughlin, D.C. & Laughlin, D.E. (2013). Advances in modeling trait-based plant community assembly. *Trends Plant Sci.* 18, 1360–1385

12.

Lavorel, S., Díaz, S., Cornelissen, J.H.C., Garnier, E., Harrison, S.P., McIntyre, S., *et al.* (2007). Plant functional types: are we getting any closer to the holy grail? In *Terrestrial Ecosystems in a Changing World* (Canadell, J.G., Pataki, D., Pitelka, L., eds), pp. 149–160, The IGBP Series, Springer-Verlag

13.

Poff, N.L., Pyne, M.I., Bledsoe, B.P., Cuhaciyan, C.C., & Carlisle, D.M. (2010). Developing linkages between species traits and multiscaled environmental variation to explore vulnerability of stream benthic communities to climate change. *J. N. Am. Benthol. Soc*. 29, 1441–1458

14.

Stuart-Smith, R.D., Bates, A.E., Lefcheck, J.S., Duffy, J.E., Baker, S.C., Thomson, R.J. *et al.* (2013). Integrating abundance and functional traits reveals new global hotspots of fish diversity. *Nature* 501, 539–542

15.

Villéger, S., Miranda, J.R., Hernández, D.F., & Mouillot, D. (2010). Contrasting changes in taxonomic vs. functional diversity of tropical fish communities after habitat degradation.*Ecol. Appl.* 20, 1512–1522
