# Supplementary material for: Functional traits, convergent evolution, and periodic tables of niches
Source: Ecol Lett. 2015 Jun 21;18(8):737–51. doi: 10.1111/ele.12462 (PMC4744997; doi:10.1111/ele.12462)
Supplement: Supplementary file 14 [file ELE-18-737-s014.docx]

**Supplemental Information 5: References from Table 1**

1.

Balon, E.K. (1975). Reproductive guilds of fishes: a proposal and definition. *J. Fish. Board* *Can.* 32, 821–864

2.

Bentlage, B., Peterson, A.T. Barve, N., & Cartwright, P. (2013). Plumbing the depths: extending ecological niche modelling and species distribution modelling in three dimensions. *Global Ecol. Biogeogr.* 22, 952–961

3.

Chao, A., Simon-Freeman, R., & Grether, G. (2013). Patterns of niche partitioning and alternative reproductive strategies in an east African dung beetle assemblage. *J. Insect. Behav.* 26, 525–539

4.

Danks, H.V. (1987). *Insect Dormancy: An Ecological Perspective.* Biological Survey of Canada, Ottawa.

5.

Elser, J.J., Fagan, W.F., Denno, R.F., Dobberfuhl, D.R., Folarin, A., Huberty, A. (2000). Elemental analysis illuminates nutritional constraints on terrestrial and freshwater food webs. *Nature* 408, 578–580

6.

Grime, J.P. (1979). *Plant Strategies and Vegetation Processes*. John Wiley & Sons

7.

Kolbe, J.J., Leal, M., Schoener, T.W., Spiller, D.A., & Losos, J.B. (2012). Founder effects persist despite adaptive differentiation: a field experiment with lizards. *Science* 335, 1086–1089

8.

MacArthur, R. & MacArthur, J.W. (1961). On bird species-diversity. *Ecology* 42, 594–598

9.

Massad, T.J., Fincher, R.M., Smilanich, A.M., & Dyer, L. (2011). A quantitative evaluation of major plant defense hypotheses, nature versus nurture, and chemistry versus ants. *Arthropod-Plant Interact.* 5, 125–139

10.

Moles, A. T., Peco, B., Wallis, I.R., Foley, W.J., Poore, A.G., Seabloom, E.W. (2013). Correlations between physical and chemical defences in plants: tradeoffs, syndromes, or just many different ways to skin a herbivorous cat? *New Phytol.* 198, 252–263

11.

Negret, B.S., Pérez, F., Markesteijn, L. Castillo, M.J., & Armesto, J.J. (2013). Diverging drought-tolerance strategies explain tree species distribution along a fog-dependent moisture gradient in a temperate rain forest. *Oecologia* 173, 625–635

12.

Orzack, S.H. & Tuljapurkar, S. (1989). Population dynamics in variable environments. VII. The demography and evolution of iteroparity. *Am. Nat.* 133, 901–923

13.

Pianka, E.R. (1966) Convexity, desert lizards, and spatial heterogeneity. *Ecology* 47, 1055–1059

14.

Pyke, C.R., Condit, R., Aguilar, S., & Lao, S. (2001). Floristic composition along a climatic gradient in a neotropical lowland forest. *J. Veg. Sci.* 12, 553–566

15.

Roff, D.A. & Fairbairn, D.J. (2007). The evolution and genetics of migration in insects. *BioScience* 57, 155–164

16.

Shertzer, K.W. & Ellner, S.P. (2002). Energy storage and the evolution of population dynamics. *J. Theoret. Biol.* 215,183-200

17.

Vanak, A.T., Fortin, D., Thaker, M., Ogden, M., Owen, C., Greatwood, S., & Slotow, R. (2013). Moving to stay in place: behavioral mechanisms for coexistence of African large carnivores. *Ecology* 94, 2619–2631

18.

Villegas-Amtmann, S., Jeglinski, J.W., Costa, D.P., Robinson, P.W., & Trillmich, F. (2013). Individual foraging strategies reveal niche overlap between endangered Galapagos pinnipeds. *PLoS One* 8, e70748
